# Bmi1 suppresses protein synthesis and promotes proteostasis in hematopoietic stem cells

**DOI:** 10.1101/gad.349917.122

**Published:** 2022-08-01

**Authors:** Rebecca J. Burgess, Zhiyu Zhao, Daisuke Nakada, Sean J. Morrison

**Affiliations:** 1 Children's Research Institute, Department of Pediatrics, University of Texas Southwestern Medical Center, Dallas, Texas 75390, USA;; 2 Department of Molecular and Human Genetics, Baylor College of Medicine, Houston, Texas 77030;; 3 Howard Hughes Medical Institute, University of Texas Southwestern Medical Center, Dallas, Texas 75390, USA

**Keywords:** polycomb, self-renewal, tissue regeneration, senescence, tumor suppressor

## Abstract

The polycomb complex component Bmi1 promotes the maintenance of stem cells in multiple postnatal tissues, partly by negatively regulating the expression of p16^Ink4a^ and p19^Arf^, tumor suppressors associated with cellular senescence. However, deficiency for p16^Ink4a^ and p19^Arf^ only partially rescues the function of *Bmi1*-deficient stem cells. We conditionally deleted *Bmi1* from adult hematopoietic cells and found that this slowly depleted hematopoietic stem cells (HSCs). Rather than inducing senescence, *Bmi1* deficiency increased HSC division. The increased cell division was caused partly by increased Aristaless-related homeobox (*ARX*) transcription factor expression, which also increased ribosomal RNA expression. However, *ARX* deficiency did not rescue HSC depletion. *Bmi1* deficiency also increased protein synthesis, protein aggregation, and protein ubiquitylation independent of its effects on cell division and p16^Ink4a^, p19^Arf^, and ARX expression. Bmi1 thus promotes HSC quiescence by negatively regulating ARX expression and promotes proteostasis by suppressing protein synthesis. This highlights a new connection between the regulation of stem cell maintenance and proteostasis.

## Abstract (precis)

In this study, Burgess et al. conditionally deleted Bmi1 from adult hematopoietic cells and found that this slowly depleted hematopoietic stem cells (HSCs) and that, rather than inducing senescence, Bmi1 deficiency increased HSC division. Overall, they found that Bmi1 promotes HSC quiescence by negatively regulating ARX expression and promoting proteostasis by suppressing protein synthesis.

Bmi1 is a polycomb repressive complex 1 (PRC1) component that negatively regulates the expression of target genes by binding and ubiquitylating methylated histones, promoting a repressive chromatin structure (Sparmann and van Lohuizen 2006; Simon and Kingston 2009). Bmi1 is required for the maintenance of stem cells in several postnatal tissues, including in the hematopoietic and nervous systems (Molofsky et al. 2003, 2005; Park et al. 2003; Bruggeman et al. 2005; Oguro et al. 2006; Lukacs et al. 2010; Robson et al. 2011; Zacharek et al. 2011). Given that Bmi1 is required by stem cells in many tissues, elucidation of the mechanisms by which it promotes stem cell function may reveal fundamental stem cell requirements.

Most studies of Bmi1 function have used *Bmi1* germline knockout mice, which have severe phenotypes in the hematopoietic and nervous systems, typically leading to the death of mice before adulthood (van der Lugt et al. 1994). This limited the ability to study Bmi1 function in adult stem cells. To address these limitations, we conditionally deleted *Bmi1* from neural stem/progenitor cells in 6-wk-old mice and observed reduced stem cell function and neurogenesis in the adult brain (Mich et al. 2014). This confirmed that Bmi1 is required by adult stem cells, though adult stem cell depletion appeared to occur much more slowly than the depletion of fetal/neonatal stem cells in germline knockout mice. Bmi1 is also required within adult leptin receptor^+^ bone marrow stromal cells to prevent inappropriate adipogenic differentiation (Hu et al. 2019; Kato et al. 2019) as well as within hematopoietic stem cells (HSCs) to preserve their reconstituting activity in irradiated mice (Yu et al. 2021).

Part of the mechanism by which Bmi1 promotes stem cell maintenance is by negatively regulating the expression of the p16^Ink4a^ and p19^Arf^ tumor suppressors, both of which are encoded at the *CDKN2a* locus (Jacobs et al. 1999). p16^Ink4a^ and p19^Arf^ inhibit cell cycle progression and promote cellular senescence by promoting Rb and p53 function (Sharpless and DePinho 1999). Consistent with this, mouse and human fibroblasts undergo premature senescence in the absence of Bmi1 (Jacobs et al. 1999; Itahana et al. 2003). Deficiency for p16^Ink4a^ and p19^Arf^ partially rescues *Bmi1* deficiency phenotypes in the nervous and hematopoietic systems, attenuating the depletion of stem cells and increasing the reconstituting activity of HSCs upon transplantation into irradiated mice (Jacobs et al. 1999; Iwama et al. 2004; Bruggeman et al. 2005; Molofsky et al. 2005; Oguro et al. 2006, 2010; Akala et al. 2008; Miyazaki et al. 2008). However, these rescues are only partial, as *Bmi1*-deficient HSCs and lymphocytes are still depleted in vivo (Jacobs et al. 1999; Bruggeman et al. 2005; Oguro et al. 2006, 2010).

Bmi1 also promotes stem cell function through additional cell-autonomous mechanisms. Partly as a consequence of increased p19^Arf^ expression, increased p53 and p21^cip1^ function contributes to the depletion of *Bmi1*-deficient stem cells (Bruggeman et al. 2005; Fasano et al. 2007; Akala et al. 2008; Miyazaki et al. 2008). Bmi1 also promotes DNA repair by ubiquitylating histone H2A/H2AX and recruiting DNA repair proteins (Facchino et al. 2010; Ismail et al. 2010; Chagraoui et al. 2011). Bmi1 regulates mitochondrial function, suppressing the generation of reactive oxygen species and DNA damage (Liu et al. 2009). Bmi1 binds to the E4f1 transcription factor, and knockdown of E4f1 partially rescues hematopoietic stem/progenitor cell function (Chagraoui et al. 2006). *Bmi1* deficiency increases the expression of gene products that promote lineage commitment, leading to premature stem cell differentiation (Oguro et al. 2010). Finally, Bmi1 negatively regulates Wnt pathway activation, and β-catenin deficiency partially rescues HSCs’ reconstituting potential (Yu et al. 2021).

To our knowledge, Bmi1 has not been shown to regulate protein synthesis, but HSCs depend on a highly regulated rate of protein synthesis. HSCs synthesize less protein per hour than other hematopoietic cells, even when controlling for differences in cell cycle status (Signer et al. 2014). Genetic changes that increase or decrease the protein synthesis rate reduce HSC function (Signer et al. 2014). A low rate of protein synthesis is required in HSCs to prevent the accumulation of unfolded proteins and proteostatic stress (Hidalgo San Jose et al. 2020). HSCs are more sensitive than restricted progenitors to proteostatic stress (van Galen et al. 2014). A slow rate of protein synthesis appears to be a fundamental requirement of adult stem cells, as stem cells in other tissues also synthesize protein more slowly than restricted progenitors (Llorens-Bobadilla et al. 2015; Blanco et al. 2016; Sanchez et al. 2016; Zismanov et al. 2016).

## Results

### Bmi1 promotes HSC quiescence and maintenance in adult mice

**Figure 1.**
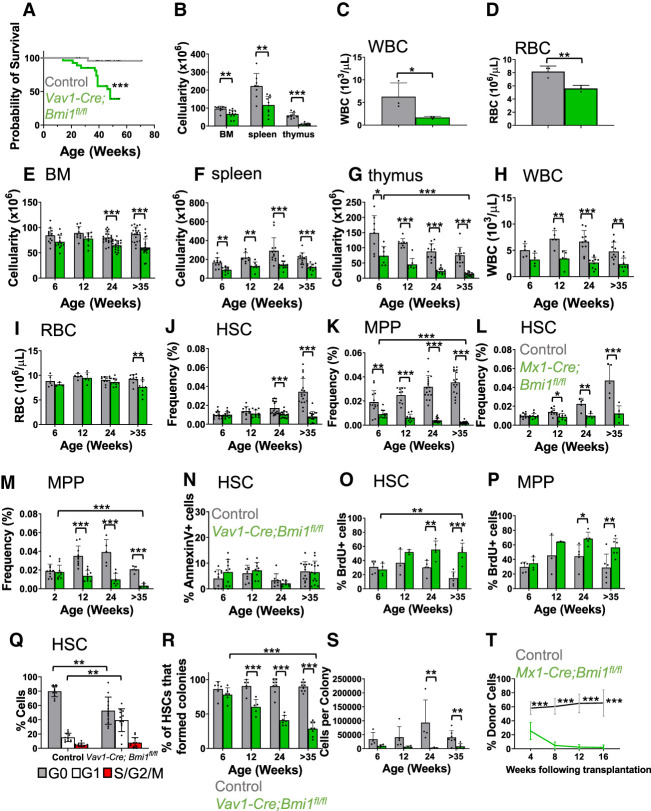
Bmi1 is required for the maintenance and quiescence of adult HSCs. (*A*) Survival curve of *Vav1-Cre; Bmi*^*fl/fl*^ and littermate control (*Bmi1*^*fl/fl*^ or *Bmi1*^*fl/+*^) mice (*n* = 25 or 27 mice per genotype). (*B*) Bone marrow (BM; two femurs and two tibias), spleen, and thymus cellularity in *Vav1-Cre; Bmi1*^*fl/fl*^ and littermate control mice at 45–55 wk of age (*n* = 8–12 mice per genotype from 10 independent experiments). (*C*,*D*) White blood cell (*C*) and red blood cell (*D*) counts in 45- to 55-wk-old mice (*n* = 3 mice per genotype from two independent experiments). (*E*–*K*) Bone marrow (*E*), spleen (*F*), and thymus (*G*) cellularity as well as WBC (*H*) and RBC (*I*) counts and HSC (*J*) and MPP (*K*) frequencies in the bone marrow of *Vav1-Cre; Bmi1*^*fl/fl*^ and littermate control mice at 6–8 wk, 12–16 wk, 24–28 wk, and >35 wk of age (*n* = 4–21 mice per genotype per time point from two to 17 independent experiments per time point). (*L*,*M*) HSC (*L*) and MPP (*M*) frequency in the bone marrow of *Mx1-Cre; Bmi1*^*fl/fl*^ and littermate control mice at 2–4 wk, 12–16 wk, 24–28 wk, and >35 wk after pIpC treatment, which was initiated at 6–8 wk of age (*n* = 5–10 mice per genotype per time point from four to nine experiments per time point). (*N*) Percentage of bone marrow HSCs that were Annexin V^+^ in *Vav1-Cre; Bmi1*^*fl/fl*^ and littermate control mice (*n* = 8–14 mice per genotype per time point from three to six experiments per time point). (*O*,*P*) Incorporation of a 72-h pulse of BrdU in HSCs (*O*) and MPPs (*P*) (*n* = 3–6 mice per genotype per time point from one to three experiments per time point). (*Q*) Percentage of HSCs in G0 (Ki67-negative and 2N DNA content), G1 (Ki67^+^ and 2N DNA content), and S/G2/M (Ki67^+^ and >2 N DNA content) in 35-wk-old *Vav1-Cre; Bmi1*^*fl/fl*^ and control mice (*n* = 11 or 12 mice per genotype from five independent experiments). (*R*,*S*) Percentage of HSCs that formed colonies in culture (*R*) and the number of cells per colony (*S*) (*n* = 3–9 mice per genotype per time point from three to seven experiments per time point). (*T*) Donor-derived CD45^+^ cells in the blood of mice competitively transplanted with *Mx1-Cre; Bmi1*^*fl/fl*^ or control donor bone marrow cells (*n* = 3 donors per genotype were transplanted into a total of 12 or 14 recipients per genotype in three independent experiments). All data represent mean ± standard deviation. (*) *P* < 0.05, (**) *P* < 0.01, (***) *P* < 0.001. Each dot reflects a different mouse. Among *Bmi1*-deficient groups, we only showed the statistical significance of differences between 6 and 35 wk.

We conditionally deleted Bmi1 in hematopoietic cells using Vav1-Cre. The *Vav1-Cre; Bmi1*^*fl/fl*^ mice died at significantly younger ages than littermate control mice, mostly before 1 yr of age (Fig. 1A). *Vav1-Cre; Bmi1*^*fl/fl*^ mice that were not overtly ill were sacrificed at 45–55 wk of age to assess hematopoiesis. They showed signs of impending hematopoietic failure, including significant reductions in bone marrow, spleen, and thymus cellularity as well as reduced white and red blood cell counts (Fig. 1B–D).

We compared hematopoiesis in *Vav1-Cre; Bmi1*^*fl/fl*^ and littermate control mice at four time points: 6–8 wk, 12–16 wk, 24–28 wk, and >35 wk of age. Bone marrow, spleen, and thymus cellularity was reduced in *Vav1-Cre; Bmi1*^*fl/fl*^ mice at most time points (Fig. 1E–G). White blood cell counts were reduced in *Vav1-Cre; Bmi1*^*fl/fl*^ mice that were at least 12 wk of age (Fig. 1H) and red blood cell counts were reduced in *Vav1-Cre; Bmi1*^*fl/fl*^ mice at 35 wk of age (Fig. 1I). In the bone marrow, *Vav1-Cre; Bmi1*^*fl/fl*^ mice had an increased frequency of Mac1^+^Gr1^+^ myeloid cells at 35 wk of age (Supplemental Fig. S1A) but no significant changes in the frequencies of Ter119^+^ erythroid lineage cells (Supplemental Fig. S1B) or CD3^+^ T cells (Supplemental Fig. S1C) as compared with littermate controls. As expected (Oguro et al. 2010; Cantor et al. 2019), *Vav1-Cre; Bmi1*^*fl/fl*^ bone marrow exhibited a significant increase in the frequency of pro-B cells (Supplemental Fig. S1D) and significant reductions in the frequencies of pre-B cells (Supplemental Fig. S1E) and B220^+^sIgM^+^ B cells (Supplemental Fig. S1F) in mice at least 12 wk of age. The depletion of bone marrow cells (Fig. 1E), thymocytes (Fig. 1G), pre-B cells (Supplemental Fig. S1E), and B220^+^sIgM^+^ B cells (Supplemental Fig. S1F) increased over time.

CD150^+^CD48^−^Lineage^−^Sca-1^+^c-kit^+^ HSCs were depleted in *Vav1-Cre; Bmi1*^*fl/fl*^ mice beginning at 24 wk of age (Fig. 1J; Supplemental Fig. S1G,H), while CD150^−^ CD48^−^Lineage^−^Sca-1^+^c-kit^+^ multipotent progenitors (MPPs) were depleted at all ages (Fig. 1K). The markers used to identify each hematopoietic stem/progenitor cell population in this study are listed in Supplemental Table S1. We observed similar depletions of HSCs and MPPs in *Mx-1-cre; Bmi1*^*fl/fl*^ mice as compared with littermate controls following treatment with polyinosine–polycytidine (pIpC) at 6 wk of age (Fig. 1L,M). *Vav1-Cre; Bmi1*^*fl/fl*^ and control mice did not significantly differ in the percentage of HSCs that were Annexin V^+^, suggesting that *Bmi1* deficiency did not induce cell death in HSCs (Fig. 1N). However, the rate at which HSCs and MPPs incorporated a 3-d pulse of 5-bromo-2′-deoxyuridine (BrdU) was significantly higher in *Vav1-Cre; Bmi1*^*fl/fl*^ as compared with control mice at 24 wk of age and older (Fig. 1O,P). Consistent with this, the percentage of Ki-67^+^ HSCs in G_1_ phase of the cell cycle was higher in *Vav1-Cre; Bmi1*^*fl/fl*^ as compared with control mice at 35 wk of age (Fig. 1Q; Supplemental Fig. S1I).

As expected, fewer *Bmi1*-deficient HSCs formed colonies in culture as compared with control HSCs, and the magnitude of the defect increased over time (Fig. 1R). *Bmi1*-deficient colonies contained fewer cells than control colonies (Fig. 1S). We confirmed by polymerase chain reaction (PCR) that *Bmi1* was recombined in 100% of the colonies formed by *Vav1-Cre; Bmi1*^*fl/fl*^ cells. Consistent with this, *Bmi1*-deficient bone marrow cells gave significantly lower levels of reconstitution than control cells upon competitive transplantation into irradiated mice (Fig. 1T; Supplemental Fig. S1J). While control bone marrow cells gave long-term multilineage reconstitution in all recipients, *Bmi1*-deficient cells did not give long-term multilineage reconstitution in any recipients, indicating reduced self-renewal potential.

### *Bmi1*-deficient HSCs do not appear to undergo senescence

**Figure 2.**
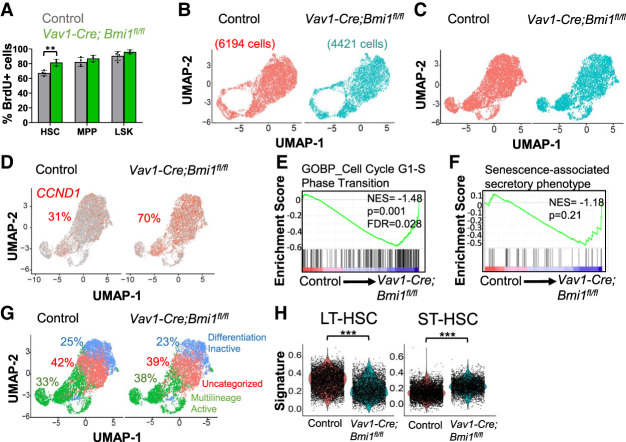
Bmi1-deficient HSCs are not senescent. (*A*) Incorporation of a 10-d pulse of BrdU into HSCs, MPPs, and LSK cells from *Vav1-Cre; Bmi1*^*fl/fl*^ and littermate control mice at 35 wk of age (*n* = 3–4 mice per genotype from two independent experiments). (*B*,*C*) UMAP plots of *Vav1-Cre; Bmi1*^*fl/fl*^ and control HSCs (25- to 32-wk-old mice) analyzed by single-cell RNA sequencing without (*B*) or with (*C*) cell cycle regression. (*D*) Percentage of Cyclin D1 (*CCND1*)*-*positive HSCs. (*E*) Enrichment of G1-to-S-phase transition genes in *Vav1-Cre; Bmi1*^*fl/fl*^ as compared with control HSCs. (*F*) No significant enrichment of senescence-associated secretory phenotype genes in *Vav1-Cre; Bmi1*^*fl/fl*^ as compared with control HSCs. (*G*) Categorization of HSCs based on markers of differentiation-inactive and multilineage-active HSCs (Pei et al. 2020). (*H*) Long-term (LT)-HSC and short-term (ST)-HSC gene expression signatures (Pei et al. 2020) in HSCs. Data represent mean ± standard deviation. (**) *P* < 0.01, (***) *P* < 0.001. Each dot reflects a different mouse (*A*) or HSC (*B*–*H*).

Since cellular senescence would reduce the percentage of dividing HSCs, the increased division of *Bmi1*-deficient HSCs (Fig. 1O) suggested that they were not depleted by cellular senescence. However, it remained possible that a subset of *Bmi1*-deficient HSCs underwent senescence while the remaining HSCs proliferated in an effort to restore homeostasis. To test this, we performed a longer-term BrdU incorporation study to test whether there was an increased frequency of nondividing HSCs in 35-wk-old *Vav1-Cre; Bmi1*^*fl/fl*^ mice. After a 10-d pulse of BrdU, 90%–96% of Lin^−^Sca-1^+^c-kit^+^ (LSK) cells and 82%–87% of MPPs were BrdU^+^ in both *Vav1-Cre; Bmi1*^*fl/fl*^ and littermate control mice (Fig. 2A). A significantly higher percentage of *Bmi1*-deficient HSCs incorporated BrdU (82% ± 4.6%) as compared with control HSCs (67% ± 3.6%). This further suggested that *Bmi1*-deficient HSCs were not undergoing senescence.

We also performed single-cell RNA sequencing of HSCs from 28- to 32-wk-old *Vav1-Cre; Bmi1*^*fl/fl*^ and control mice. Following sequencing and quality control analysis, we analyzed gene expression in a total of 6194 control cells and 4421 *Bmi1*-deficient HSCs, with a mean number of genes detected per cell of 3068 in control and 4031 in *Bmi1*-deficient HSCs. Cluster analysis, with (Fig. 2C) or without (Fig. 2B) cell cycle regression, did not separate control and *Bmi1*-deficient HSCs into distinct clusters, and we did not detect a transcriptionally distinct subpopulation of *Bmi1*-deficient HSCs that was not present in control HSCs. We found that 70% of *Bmi1*-deficient and 31% of control HSCs were positive for *cyclin D1* (*CCND1*) (Fig. 2D), a gene expressed primarily in G1 phase of the cell cycle (Passegué et al. 2005). There was no cluster of *Bmi1*-deficient HSCs that lacked cells with *CCND1* expression, and the clusters with fewer *CCND1*-expressing cells were present in both *Bmi1*-deficient and control HSCs. Consistent with the increases in *CCND1* expression and BrdU incorporation (Fig. 1O), *Bmi1-*deficient HSCs were enriched for the expression of G_1_-to-S-phase cell cycle transition genes (Fig. 2E). Together, these observations suggest there is not a distinct subset of postmitotic *Bmi1*-deficient HSCs, as would be expected if the cells were undergoing senescence.

Senescence is also marked by a senescence-associated secretory phenotype (SASP). We performed gene set enrichment analysis using a curated list of SASP genes (Liu et al. 2019) and found that SASP genes were not significantly enriched in *Bmi1*-deficient HSCs (Fig. 2F). We also performed cluster identification using markers of differentiation-inactive and multilineage-active HSCs (Pei et al. 2020). We observed similar percentages of differentiation-inactive and multilineage-active HSCs among *Bmi1*-deficient and control HSCs (Fig. 2G).

If *Bmi1*-deficient HSCs did not undergo senescence, they may have been depleted by premature lineage commitment (Oguro et al. 2010). Consistent with this possibility, we found that *Bmi1*-deficient HSCs were less enriched than control HSCs for a long-term HSC transcriptional signature and more enriched than control HSCs for a short-term HSC transcriptional signature (Fig. 2H). This suggests that *Bmi1*-deficient HSCs exited the stem cell pool more rapidly than control HSCs without completely losing the ability to divide or to form differentiated progeny.

### *p16*^*Ink4a*^ and *p19*^*Arf*^ deficiency does not rescue the depletion of *Bmi1*-deficient HSCs

**Figure 3.**
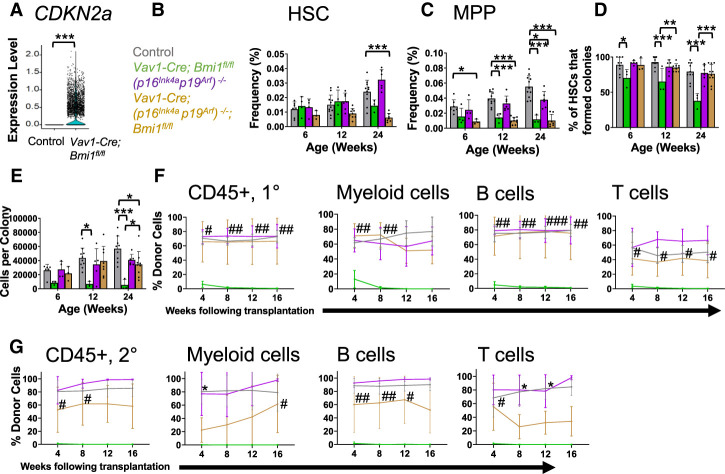
Deficiency for *p16*^*Ink4a*^ and *p19*^*Arf*^ partially rescues the function of *Bmi1*-deficient HSCs but does not prevent their depletion. (*A*) Expression of *CDKN2a* in single HSCs from *Vav1-Cre; Bmi1*^*fl/fl*^ and control mice. (*B*,*C*) Frequencies of HSCs (*B*) and MPPs (*C*) in the bone marrow of control, *Vav1-Cre; Bmi1^fl/fl^*,* (p16^Ink4a^p19^Arf^)*^−*/*−^, and *Vav1-Cre; (p16^Ink4a^p19^Arf^)*^−*/*−^*; Bmi1*^*fl/fl*^ mice (*n* = 3–11 mice per genotype per timepoint from four to eight independent experiments per time point). (*D*,*E*) Percentage of HSCs that formed colonies (*D*) and number of cells per colony (*E*) (*n* = 3–11 mice per genotype per time point from four to eight experiments per time point). (*F*) Donor contribution to CD45^+^ cells, Mac-1^+^Gr-1^+^ myeloid cells, B220^+^ B cells, and CD3^+^ T cells in the blood of mice competitively transplanted with donor bone marrow cells from control, *Vav1-Cre; Bmi1^fl/fl^*,* (p16^Ink4a^p19^Arf^)*^−*/*−^, or *Vav1- Cre; (p16^Ink4a^p19^Arf^)*^−*/*−^*; Bmi1*^*fl/fl*^ mice (*n* = 2 donors per genotype transplanted into a total of five to nine recipients per genotype in two independent experiments). (*G*) Donor contribution to CD45^+^ cells, Mac-1^+^Gr-1^+^ myeloid cells, B220^+^ B cells, and CD3^+^ T cells in the blood of secondary recipients transplanted with bone marrow cells from the primary recipients in *F* (*n* = 3–4 donors per genotype transplanted into eight to 10 recipients per donor in two independent experiments). All data represent mean ± standard deviation. (*) *P* < 0.05, (**) *P* < 0.01, (***) *P* < 0.001. Each dot reflects an HSC (*A*) or a different mouse (*B*–*G*). (*F*,*G*) In the figure panels that show reconstitution assays, we only show the statistical significance of differences between mice reconstituted by control (*) or *Bmi1*-deficient (#) cells versus double-mutant cells.

As expected, *p16*^*Ink4a*^ and *p19*^*Arf*^ expression was increased in *Bmi1*-deficient as compared with control HSCs (Supplemental Fig. S2A,B). By single-cell RNA sequencing, *CDKN2a* transcripts (which encode both gene products) were not detected in control HSCs but were detected in 42% of *Bmi1*-deficient HSCs (Fig. 3A). To test whether *p16*^*Ink4a*^ deficiency rescued *Bmi1* deficiency phenotypes, we generated *Vav1-Cre; p16*^*Ink4a*^^-*fl/fl*^*; Bmi1*^*fl/fl*^ double-mutant mice and compared hematopoiesis in these mice with control, *Vav1-Cre; p16*^*Ink4a*^^-*fl/fl*^, and *Vav1-Cre; Bmi1*^*fl/fl*^ single-mutant mice. *p16*^*Ink4a*^ deficiency by itself did not significantly affect HSC frequency (Supplemental Fig. S2C), MPP frequency (Supplemental Fig. S2D), the percentage of HSCs that formed colonies in culture (Supplemental Fig. S2E), the cellularity of those colonies (Supplemental Fig. S2F), or the capacity of bone marrow cells to give long-term multilineage reconstitution upon competitive transplantation into irradiated mice (Supplemental Fig. S2G). *Bmi1* deficiency significantly reduced all of these parameters by 35 wk of age, and *p16*^*Ink4a*^ deficiency did not significantly rescue any of these phenotypes with the exception of a partial rescue of the cellularity of colonies formed by HSCs in culture (Supplemental Fig. S2C–G).

We also generated *Vav1-Cre; p19*^*Arf*^^-*fl/fl*^*; Bmi1*^*fl/fl*^ double-mutant mice. *p19*^*Arf*^ deficiency by itself did not significantly affect HSC or MPP frequency (Supplemental Fig. S2H,I) and had little or no effect on the percentage of HSCs that formed colonies in culture (Supplemental Fig. S2J), colony cellularity (Supplemental Fig. S2K), or the capacity of bone marrow cells to give long-term multilineage reconstitution upon competitive transplantation (Supplemental Fig. S2L). *Bmi1* deficiency significantly reduced all of these parameters by 24 wk of age (Supplemental Fig. S2H–L). *p19*^*Arf*^ deficiency partially rescued the reconstituting activity of *Bmi1*-deficient bone marrow cells (Supplemental Fig. S2L) but did not rescue myeloid reconstitution or any other *Bmi1* deficiency phenotypes (Supplemental Fig. S2H–L). Upon serial transplantation of bone marrow cells from the primary recipients into secondary recipient mice, *p19*^*Arf*^ deficiency did not rescue the reconstituting activity of *Bmi1*-deficient bone marrow cells (Supplemental Fig. S2M).

Finally, we generated *Vav1-Cre; (p16^Ink4a^p19^Arf^)*^−*/*−^*; Bmi1*^*fl/fl*^ triple-mutant mice that lacked both *p16*^*Ink4a*^ and *p19*^*Arf*^. *p16*^*Ink4a*^ and *p19*^*Arf*^ deficiency did not rescue the effect of *Bmi1* deficiency on HSC frequency (Fig. 3B) or MPP frequency (Fig. 3C) but did rescue the effects of *Bmi1* deficiency on the percentage of HSCs that formed colonies in culture (Fig. 3D), the cellularity of those colonies (Fig. 3E), and the reconstituting capacity of bone marrow cells in irradiated mice (Fig. 3F). Upon serial transplantation of those bone marrow cells into secondary recipient mice, *p16*^*Ink4a*^ and *p19*^*Arf*^ deficiency partially rescued the reconstituting activity of *Bmi1*-deficient bone marrow cells (Fig. 3G). These data show that *p16*^*Ink4a*^ and *p19*^*Arf*^ deficiency partially rescues the effects of *Bmi1* deficiency on HSC function but does not fully rescue HSC depletion. Therefore, we sought to identify additional mechanisms by which Bmi1 promotes HSC maintenance.

### ARX expression promotes the proliferation of *Bmi1*-deficient HSCs

We compared *Bmi1*-deficient and control HSCs by bulk RNA sequencing. Differentially expressed genes identified by bulk RNA sequencing (FDR < 0.05, fold change > 2) and single-cell RNA sequencing (FDR < 0.05, fold change > 2) were significantly correlated (*R*^2^= 0.73, *P* < 0.001). In the bulk RNA sequencing analysis, six genes were significantly more highly expressed by *Bmi1*-deficient as compared with control HSCs and 12 genes were expressed at lower levels in *Bmi1*-deficient HSCs (FDR < 0.05, fold change > 3) (Supplemental Table S2). The two genes that were most highly up-regulated in *Bmi1*-deficient HSCs were the Aristaless-related homeobox (*ARX*; 34-fold) transcription factor and *CDKN2a* (9.3-fold). ARX promotes the proliferation, migration, and differentiation of neural (Kitamura et al. 2002; Fulp et al. 2008; Colasante et al. 2015), muscle (Biressi et al. 2008), and pancreatic (Collombat et al. 2003) progenitors.

**Figure 4.**
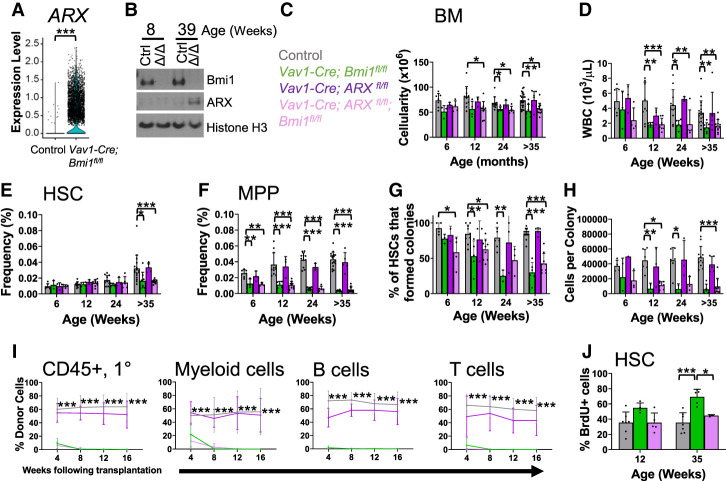
Bmi1 promotes HSC quiescence partly by negatively regulating ARX expression. (*A*) *ARX* expression in single HSCs from *Vav1-Cre; Bmi1*^*fl/fl*^ and control mice. (*B*) Western blot of ARX protein in *Vav1-Cre; Bmi1*^*fl/fl*^ (Δ/Δ) versus control (Ctrl) LSK cells. (*C*–*F*) Bone marrow cellularity (*C*), white blood cell counts (*D*), and HSC (*E*) and MPP (*F*) frequency in the bone marrow of control, *Vav1-Cre; Bmi1^fl/fl^, Vav1-Cre; ARX*^*fl/fl*^, and *Vav1-Cre; ARX^fl/fl^; Bmi1*^*fl/fl*^ mice at the indicated ages (*n* = 3–22 mice per genotype per time point from four to 12 experiments per time point). (*G*,*H*) Percentage of HSCs that formed colonies (*G*) and the number of cells per colony (*H*) (*n* = 1–12 mice per genotype per time point from three to eight independent experiments per time point). (*I*) Donor-derived CD45^+^ hematopoietic cells, Mac-1^+^Gr-1^+^ myeloid cells, B220^+^ B cells, and CD3^+^ T cells in the blood of mice competitively transplanted with control, *Vav1-Cre; Bmi1*^*fl/fl*^, *Vav1-Cre; ARX*^*fl/fl*^, and *Vav1-Cre; ARX^fl/fl^; Bmi1*^*fl/fl*^ donor bone marrow cells. Donors were 6–8 wk of age (*n* = 2–3 donors/genotype transplanted into a total of six to 11 recipients per genotype in three independent experiments). Only the statistical significance of differences between *Vav1-Cre; ARX^fl/fl^; Bmi1*^*fl/fl*^ and control are shown. (*J*) Incorporation of a 72-h pulse of BrdU into HSCs from control, *Vav1-Cre; Bmi1*^*fl/fl*^, and *Vav1-Cre; ARX^fl/fl^; Bmi1*^*fl/fl*^ mice (*n* = 3–7 mice per genotype per time point from four to seven independent experiments per time point). Data represent mean ± standard deviation. (*) *P* < 0.05, (**) *P* < 0.01, (***) *P* < 0.001. Each dot reflects an HSC (*A*) or a different mouse (*B*–*J*).

Consistent with these results from bulk RNA sequencing, single-cell RNA sequencing showed that *ARX* was expressed by most (69%) *Bmi1*-deficient HSCs but rarely by control HSCs (0.2%) (Fig. 4A). Western blots showed no detectable ARX protein in control LSK cells or LSK cells from 8-wk-old *Vav1-Cre; Bmi1*^*fl/fl*^ mice (Fig. 4B) when HSC depletion was not observed (Fig. 1J). However, ARX protein was detected in LSK cells from 39-wk-old *Vav1-Cre; Bmi1*^*fl/fl*^ mice (Fig. 4C) when HSC depletion was observed (Fig. 1J). *ARX* was preferentially expressed by HSCs and MPPs as compared with differentiated cells from *Vav1-Cre; Bmi1*^*fl/fl*^ mice (Supplemental Fig. 3A).

To test whether ARX expression contributed to defects in HSC function, we generated a floxed allele to conditionally delete the C-terminal end of *ARX*, disrupting the nuclear localization sequence and the β-catenin binding domain (Supplemental Fig. S3B,C; Cho et al. 2017). We found no gross hematopoietic defects in *Vav1-Cre; ARX*^*fl/fl*^ as compared with control mice (Supplemental Fig. S3D–K), consistent with our inability to detect ARX expression in normal HSCs. To test whether *ARX* deficiency rescued *Bmi1* deficiency phenotypes, we compared hematopoiesis in *Vav1-Cre; ARX^fl/fl^; Bmi1*^*fl/fl*^ double-mutant mice with *Vav1-Cre; ARX*^*fl/fl*^ and *Vav1-Cre; Bmi1*^*fl/fl*^ single-mutant mice. *ARX* deficiency by itself did not significantly affect bone marrow cellularity (Fig. 4C), white blood cell counts (Fig. 4D), HSC frequency (Fig. 4E), MPP frequency (Fig. 4F), the percentage of HSCs that formed colonies in culture (Fig. 4G), the cellularity of those colonies (Fig. 4H), or the capacity of bone marrow cells to give long-term multilineage reconstitution upon competitive transplantation into irradiated mice (Fig. 4I). *Bmi1* deficiency significantly reduced all of these parameters by 35 wk of age and *ARX* deficiency did not significantly rescue any of these *Bmi1* deficiency phenotypes (Fig. 4C–I). However, *ARX* deficiency did rescue the increased rate of cell division in *Bmi1*-deficient HSCs in 35-wk-old mice (Fig. 4J). ARX expression thus contributed to the increased division of *Bmi1*-deficient HSCs.

### Bmi1 promotes proteostasis

**Figure 5.**
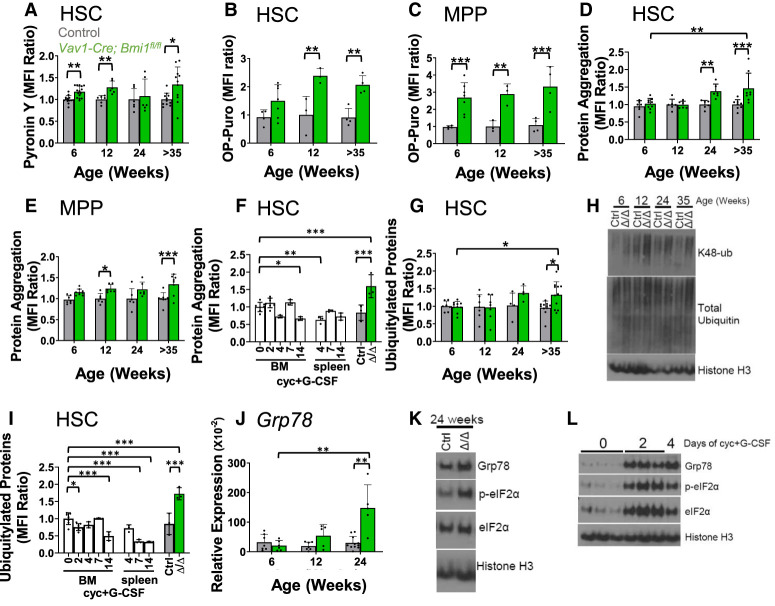
Bmi1 regulates protein synthesis and proteostasis in HSCs. (*A*) Pyronin Y staining of RNA in *Vav1-Cre; Bmi1*^*fl/fl*^ and control HSCs at 6–8 wk, 12–16 wk, 24–28 wk, and >35 wk of age (*n* = 6–15 mice per genotype per time point from two to five independent experiments per time point). (*B*,*C*) Incorporation of a 1-h pulse of OP-Puro into HSCs (*B*) and MPPs (*C*) from *Vav1-cre; Bmi1*^*fl/fl*^ and control mice (*n* = 3–7 mice per genotype per time point from one to two experiments per time point). (*D*,*E*) Proteostat dye staining in HSCs (*D*) and MPPs (*E*) (*n* = 6–9 mice per genotype per time point from two to three experiments per time point). (*F*) Proteostat dye staining in HSCs following cyclophosphamide (Cyc) plus G-CSF treatment for 2, 4, 7, or 14 d. Untreated *Vav1-Cre; Bmi1*^*fl/fl*^ (Δ/Δ) and control (Ctrl) mice at 24–38 wk of age were included in the analysis (*n* = 3*–*7 mice per treatment from three experiments). (*G*) Polyubiquitylated and monoubiquitylated protein levels in permeabilized HSCs by flow cytometry (*n* = 4–11 mice per genotype per time point from two to six independent experiments per time point). (*H*) K48-linked ubiquitin and total ubiquitin levels in LSK cells from *Vav1-Cre; Bmi1*^*fl/fl*^ (Δ/Δ) and control (Ctrl) mice analyzed by Western blot. (*I*) Polyubiquitylated and monoubiquitylated protein levels in permeabilized HSCs following cyclophosphamide (Cyc) plus G-CSF treatment (*n* = 3–7 mice per treatment from three experiments). (*J*) qRT-PCR analysis of *GRP78* levels in HSCs (*n* = 5–11 mice per genotype per time point from six independent experiments per time point). (*K*) Western blot of GRP78, phospho-eIF2α, and total eIF2α levels in LSK cells from *Vav1-Cre; Bmi1*^*fl/fl*^ (Δ/Δ) and control (Ctrl) mice. (*L*) Western blot of GRP78, phospho-eIF2α, and total eIF2α levels in LSK cells from the bone marrow of untreated control mice or mice treated with Cyc plus G-CSF for 2 or 4 d. All data represent mean ± standard deviation. (*) *P* < 0.05, (**) *P* < 0.01, (**) *P* < 0.001. Each dot reflects a different mouse. (*A–J*) Among *Bmi1*-deficient groups, we only showed the statistical significance of differences between 6 and 35 wk.

We performed gene ontology (GO) term enrichment analysis on genes that were significantly more highly expressed by *Bmi1*-deficient as compared with control HSCs (Table 1). They included several GO terms related to ribosome biogenesis, translation, unfolded protein response, and misfolded proteins. This raised the possibility that Bmi1 regulates proteostasis in HSCs. To explore this, we stained HSCs from 6- to 35-wk-old *Vav1-Cre; Bmi1*^*fl/fl*^ and control mice with Pyronin Y, which stains RNA, 80%–90% of which is ribosomal (Eun 1996). Pyronin Y staining was significantly higher in *Bmi1*-deficient as compared with control HSCs at most time points (Fig. 5A; Supplemental Fig. S4A). Consistent with this, in the bulk RNA sequencing, 25 genes that encode ribosome protein subunits (out of a total of 87 ribosome protein subunits that were expressed) were significantly more highly expressed (*P* < 0.05, fold change > 1.5) in *Bmi1*-deficient as compared with control HSCs at 52 wk after *Bmi1* deletion (BioProject ID: PRJNA834838). Bmi1 thus negatively regulates the expression of ribosomal subunits.

**Table 1.**
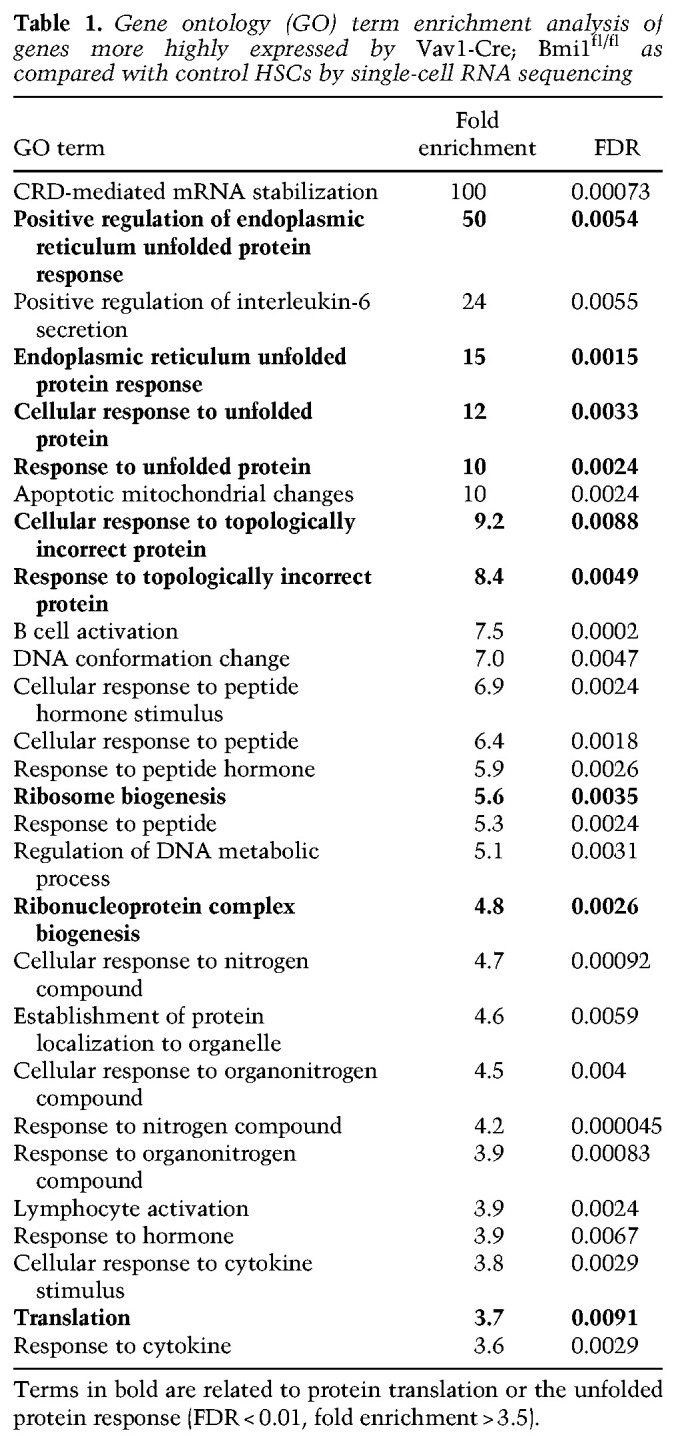
Gene ontology (GO) term enrichment analysis of genes more highly expressed by *Vav1-Cre; Bmi1*^*fl/fl*^ as compared with control HSCs by single-cell RNA sequencing

To test whether protein synthesis was increased in *Bmi1*-deficient as compared with control HSCs, we administered O-propargyl-puromycin (OP-Puro) (Signer et al. 2014) to *Vav1-Cre; Bmi1*^*fl/fl*^ and littermate control mice. *Bmi1*-deficient HSCs exhibited significantly higher levels of OP-Puro incorporation than control HSCs at 12 and 35 wk of age (Fig. 5B; Supplemental Fig. S4B). *Bmi1*-deficient MPPs exhibited significantly higher levels of OP-Puro incorporation as compared with control MPPs at all ages (Fig. 5C). The magnitude of the increases in protein synthesis in *Bmi1*-deficient HSCs (approximately twofold) would be expected to promote HSC depletion (Signer et al. 2014) as a consequence of the accumulation of misfolded proteins (Hidalgo San Jose et al. 2020).

To test whether *Bmi1*-deficient HSCs exhibited increased protein aggregation, we stained HSCs and MPPs with Proteostat, a dye that stains protein aggregates (Chapple et al. 2018). *Bmi1*-deficient HSCs exhibited significantly higher levels of Proteostat staining than control HSCs at 24 and 35 wk of age (Fig. 5D; Supplemental Fig. S4C). *Bmi1*-deficient MPPs also exhibited significantly higher levels of Proteostat staining as compared with control MPPs at multiple time points (Fig. 5E). Proteostat staining thus increased in *Bmi1*-deficient HSCs when HSC depletion was observed (Fig. 1J). This increase in protein aggregates within *Bmi1*-deficient HSCs did not appear to have been caused by increased cell division because when HSC division was induced by treatment with cyclophosphamide followed by 2, 4, 7, or 14 d of granulocyte colony-stimulating factor (G-CSF) treatment (Morrison et al. 1997), Proteostat staining did not significantly increase in bone marrow or spleen HSCs from these mice as compared with control mice (Fig. 5F).

Levels of ubiquitylated proteins increase in HSCs with proteostatic stress, presumably as a result of increased proteasome activity (Hidalgo San Jose et al. 2020). We fixed and permeablized HSCs from *Vav1-Cre; Bmi1*^*fl/fl*^ and control mice and quantitated ubiquitylated proteins by flow cytometry (Hidalgo San Jose et al. 2020). *Bmi1-*deficient HSCs exhibited significantly higher levels of polyubiquitylated and monoubiquitylated proteins as compared with control HSCs at 35 wk of age, and the levels of these ubiquitylated proteins appeared to increase over time in *Bmi1*-deficient HSCs (Fig. 5G; Supplemental Fig. S4D). By Western blot, we also observed an increase in the levels of lysine 48-linked polyubiquitin chains (K48-ub), which mark proteins for proteasome degradation (Fig. 5H; Chau et al. 1989). Conversely, total ubiquitin levels did not clearly differ between *Bmi1*-deficient and control LSK cells (Fig. 5H). The increase in ubiquitylated proteins within *Bmi1*-deficient HSCs did not appear to have been caused by increased cell division because when HSC division was induced by treatment with cyclophosphamide plus G-CSF, ubiquitylated protein levels did not significantly increase in HSCs (Fig. 5I). *Bmi1* deficiency thus increased protein aggregation and ubiquitylation independent of its effects on cell cycle.

Consistent with these markers of proteostatic stress, *Bmi1*-deficient HSCs also expressed markers of an unfolded protein response. By qRT-PCR, we observed increased levels of *Grp78/BiP* in *Bmi1*-deficient as compared with control HSCs at 24 wk of age (Fig. 5J). By Western blot, we observed increased levels of Grp78/BiP and phosphorylated eIF2α in *Bmi1*-deficient as compared with control LSK cells at 24 wk of age (Fig. 5K). Induction of HSC division by treatment with cyclophosphamide plus G-CSF was also sufficient to increase the levels of Grp78/BiP, phosphorylated eIF2α, and total eIF2α (Fig. 5L). Therefore, it is not clear to what extent the unfolded protein response markers induced in *Bmi1*-deficient HSCs were a consequence of increased cell division, protein aggregation independent of cell division, or both.

### Bmi1 regulates ARX, protein synthesis, and proteostasis independently of p16^Ink4a^/p19^Arf^

**Figure 6.**
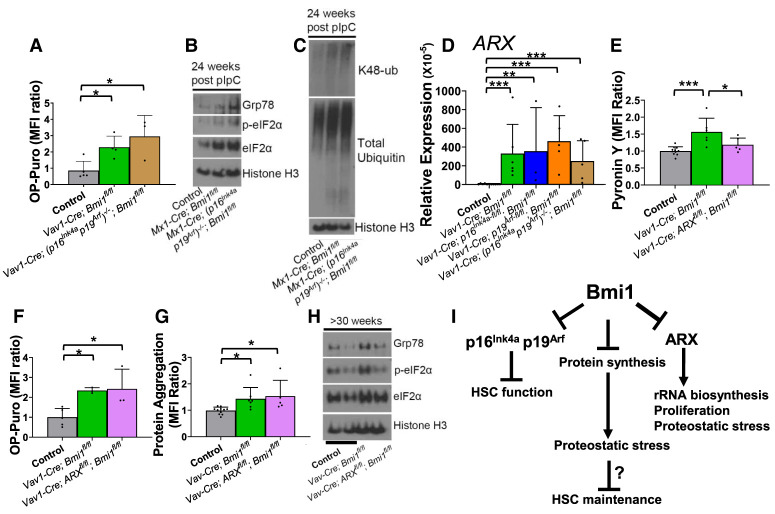
Bmi1 regulates protein synthesis and proteostasis independently of p16^Ink4a^/p19^Arf^ and ARX. (*A*) Incorporation of a 1-h pulse of OP-Puro into HSCs in 24- to 35-wk-old control, *Vav1-Cre; Bmi1*^*fl/fl*^, and *Vav1-Cre; (p16^Ink4a^p19^Arf^)*^−*/*−^*; Bmi1*^*fl/fl*^ mice (*n* = 3–5 mice per genotype from two experiments). (*B*,*C*) Western blot of GRP78, phospho-eIF2α, and total eIF2α levels (*B*) or K48-linked ubiquitin and total ubiquitin levels (*C*) in LSK cells from control, *Mx1-Cre; Bmi1*^*fl/fl*^, and *Mx1-Cre; (p16^Ink4a^ p19^Arf^)*^−*/*−l^*; Bmi1*^*fl/fl*^ mice at 24 wk after pIpC treatment. (*D*) qRT-PCR analysis of *ARX* levels in HSCs isolated from mice of the indicated genotypes at 24–28 wk of age (*n* = 3–11 mice per genotype from three to six independent experiments per genotype). (*E*–*H*) Pyronin Y staining (*E*), OP-Puro incorporation (*F*), and Proteostat dye staining (*G*) in HSCs and Western blot analysis of GRP78, phospho-eIF2α, and total eIF2α levels (*H*) in LSK cells from control, *Vav1-Cre; Bmi1*^*fl/fl*^, and *Vav1-Cre; ARX^fl/fl^; Bmi1*^*fl/fl*^ mice at 35 wk of age (*n* = 3–11 mice per genotype from three to six independent experiments). (*I*) Model summarizing the data in this study. All data represent mean ± standard deviation. (*) *P* < 0.05, (**) *P* < 0.01, (***) *P* < 0.001. Each dot reflects a different mouse.

Deficiency for *p16*^*Ink4a*^ and *p19*^*Arf*^ did not rescue the increases in protein synthesis (Fig. 6A), Grp78/BiP or phosphorylated eIF2α levels (Fig. 6B), lysine 48-linked polyubiquitylated protein levels (Fig. 6C), or *ARX* expression (Fig. 6D) in *Bmi1*-deficient HSCs. Also, *ARX* deficiency did not reduce the levels of p16^Ink4a^ or p19^Arf^ expression in *Bmi1*-deficient bone marrow (Supplemental Fig. S4E). Therefore, Bmi1 regulated *ARX* expression independently of p16^Ink4a^ and p19^Arf^ expression, and the increases in protein synthesis and proteostatic stress in *Bmi1*-deficient stem/progenitor cells were not caused by increased p16^Ink4a^ and p19^Arf^ expression.

*ARX* deficiency partially rescued the increase in Pyronin Y staining within *Bmi1*-deficient HSCs (Fig. 6E) but not the increases in protein synthesis (Fig. 6F) or protein aggregation (Fig. 6G). *ARX* deficiency also rescued the increases in Grp78/BiP and phosphorylated eIF2α in *Bmi1*-deficient LSK cells (Fig. 6H). Thus, *ARX* expression contributed to the increases in ribosomal RNA (rRNA) expression, proteostatic stress, and cell division (Fig. 4J) in *Bmi1*-deficient HSCs but not the increases in protein synthesis or protein aggregation. These data further suggest that the increase in protein synthesis in *Bmi1*-deficient HSCs is not caused by the increase in cell division.

## Discussion

In both neural stem cells (Mich et al. 2014) and HSCs (Fig. 1J), conditional deletion of *Bmi1* during adulthood leads to a slow depletion of stem cells over a period of months. This is in contrast to the much more rapid stem cell depletion in germline *Bmi1*-deficient mice (Park et al. 2003; Oguro et al. 2006; Akala et al. 2008) and the more rapid depletion of mature B cells following deletion of *Bmi1* in adult hematopoietic cells (Supplemental Fig. S1F). Our data suggest that the slow depletion of *Bmi1*-deficient HSCs in adults is not due to the induction of cellular senescence (Fig. 2) despite the fact that *Bmi1* deficiency causes senescence in fibroblasts (Jacobs et al. 1999; Itahana et al. 2003). *Bmi1*-deficient HSCs divide more frequently than control HSCs (Fig. 1O), and we were unable to detect a subset of HSCs that were transcriptionally distinct from control HSCs (Fig. 2C,G). *Bmi1* deficiency may promote HSC depletion partly as a result of premature lineage restriction (Oguro et al. 2010; Nitta et al. 2020). However, *Bmi1*-deficient HSCs also exhibited increased protein synthesis (Fig. 5B), proteostatic stress (Fig. 5), and accumulation of protein aggregates (Fig. 5D) as compared with control HSCs. Proteostatic stress increased over time after *Bmi1* deletion. HSCs are unusually sensitive to proteotoxic stress (Signer et al. 2014; Hidalgo San Jose et al. 2020), which can promote cell death (van Galen et al. 2014, 2018) or differentiation (Vilchez et al. 2012). Thus, Bmi1 appears to promote adult HSC maintenance partly by promoting proteostasis (Fig. 6I).

Bmi1 regulates HSC function by directly or indirectly negatively regulating ARX expression. The Bmi1-containing PRC1 complex represses target genes by promoting histone H2A ubiquitylation (H2AK119ub) (Cao et al. 2005). Both Bmi1 and H2AK119ub are enriched within the *ARX* promoter in LSK cells from Bmi1-overexpressing mice, raising the possibility that Bmi1 directly represses ARX expression (Nitta et al. 2020). In *Bmi1*-deficient mice, increased ARX expression promoted HSC proliferation (Fig. 4J), consistent with the known role of ARX in promoting the proliferation of fetal stem/progenitor cells in other tissues (Kitamura et al. 2002; Collombat et al. 2003; Biressi et al. 2008; Fulp et al. 2008; Colasante et al. 2015). While *ARX* deficiency rescued the expression of some markers of proteostatic stress (Fig. 6H), it did not rescue the depletion of *Bmi1*-deficient HSCs (Fig. 4E,I) or the increases in protein synthesis (Fig. 6F) or protein aggregation (Fig. 6G). This suggests that ARX contributes to proteostatic stress in *Bmi1*-deficient HSCs but that other mechanisms also contribute.

While the increases in protein aggregation and protein ubiquitylation in *Bmi1*-deficient HSCs could not be explained by increased cell division (Fig. 5F,I), Grp78/BiP and phosphorylated eIF2α levels did increase upon the induction of cell division (Figs. 5L, 6H); therefore, the proteostatic stress in *Bmi1*-deficient HSCs appears to reflect both increased cell division and effects of *Bmi1* deficiency independent of cell division. The increased levels of phosphorylated eIF2α in *Bmi1*-deficient HSCs may attenuate the increased protein synthesis in these cells (Harding et al. 1999) without completely restoring normal rates of protein synthesis.

In contrast to the increase in ribosomal gene expression in *Bmi1*-deficient HSCs, *Bmi1* deficiency reduces the expression of ribosomal genes in erythroid progenitors (Gao et al. 2015). In HSCs, Bmi1 appears to negatively regulate ribosomal gene expression by directly or indirectly inhibiting ARX expression (Fig. 6E), though the degree to which increased ribosome gene expression contributes to the increases in protein synthesis and proteostatic stress are unclear. The effects of Bmi1 on oxidative stress (Liu et al. 2009) and DNA damage could also potentially contribute to the effects on protein synthesis and proteostasis (Lee et al. 2018). Together, these data raise the possibility that the regulation of protein synthesis and proteostasis is a characteristic broadly shared among stem cell self-renewal regulators.

## Materials and methods

### Mice

*ARX*^*fl*^ mice were generated by the University of Texas Southwestern Medical Center Transgenic Technology Center by microinjecting into C57BL embryos sgRNAs against sequences flanking the region to be targeted (Integrated DNA Technologies) (Supplemental Table S3) as well as a donor oligo encoding exon 3 flanked by loxp sites (Genewiz) (Supplemental Table S3). Chimeric mice were genotyped by sequencing the targeted locus and by polymerase chain reaction analysis. Founders were backcrossed with C57Bl/Ka mice for at least three generations prior to analysis. *Mx1-Cre* mice (Kühn et al. 1995), *Vav1-Cre* mice (de Boer et al. 2003), *p16*^*Ink4a-fl*^ mice (Qiu et al. 2011), and *p19*^*Arf-fl*^ mice (Gromley et al. 2009) were obtained from Jackson Laboratory. (*p16^Inka^p19*^*Arf*^)*^+/−^* embryos (Serrano et al. 1996) were obtained from the National Cancer Institute repository, and mice were generated using the University of Texas Southwestern Medical Center Transgenic Technology Center. Mice were backcrossed with C67BL/Ka five generations before using for experiments. *Bmi1*^*fl*^ were previously described (Mich et al. 2014). Mice of the indicated ages were used, male and female in all experiments, including littermate or age-matched controls. In some experiments, (*p16*^*Inka*^*p19*^*Arf*^)*^+/−^* mice were used as controls. To induce Cre expression in *Mx1-Cre* mice, both *Mx1-Cre* and control animals received an intraperitoneal injection of 20 µg of polyinosinic:polycytidylic acid (pIpC; GE Healthcare) in PBS every other day for three to five injections starting at 5–6 wk of age. For cyclophosphamide and G-CSF injections, 4 mg of cyclophosphamide was administered by intraperitoneal injection on day 0, and 5 µg of G-CSF (Neupogen, Amgen) was administered by subcutaneous injection on each of two to 14 subsequent days, with endpoint analysis the next day. C57BL/Ka-Thy-1.2 (CD45.1) and C57BL/Ka-Thy-1.1/Thy-1.2 (CD45.1/C45.2) mice were used as recipient mice and a source of competitor bone marrow cells in transplantation experiments. All mice were housed in AAALAC-accredited, specific pathogen-free animal care facilities at University of Texas Southwestern Medical Center. All procedures were approved by the University of Texas Southwestern Medical Center Institutional Animal Care and Use Committee.

### Flow cytometric analysis and sorting of hematopoietic cells

Bone marrow from two tibias and two femurs was flushed using staining medium (Ca^2+^- and Mg^2+^-free HBSS supplemented with 2% heat-inactivated bovine serum) and dissociated into a single-cell suspension by gentle trituration with a 23-gauge needle. To sort HSCs, bone marrow was obtained from tibias, femurs, pelvises, and spines crushed using a mortar and pestle. Spleen and thymus were mechanically dissociated by crushing between two glass slides followed by trituration. Cells were resuspended in staining medium and filtered through 90-µm nylon mesh. Each cell population was identified by staining with antibodies (Supplemental Table S4) against the surface markers shown in Supplemental Table S1. Cells were counted and stained with antibodies for 30 min at 4°C. All antibodies were used at 1:400 dilution. To isolate HSCs, cells were first stained with biotin-conjugated c-kit antibody followed by incubation with antibiotin paramagnetic microbeads (Miltenyi Biotec) and streptavidin-APC780. c-kit-positive cells were enriched using LS columns or AutoMACs Pro separator (Militenyi Biotec). Lineage markers included antibodies against CD2, CD3, CD5, CD8a, Gr1, Ter119, and B220. Cells were analyzed on a FACS Canto RUO (BD Biosciences) or FACSAria II SORP (BD Biosciences) cytometer. Dead cells were identified and eliminated from analyses by including 1 µg/mL 4′,6-diamidino-2-phenylindole (DAPI) in the staining medium. Flow cytometry data were analyzed using FlowJo (BD Biosciences). Annexin V staining was performed using Annexin V APC (Thermo Fisher Scientific).

For peripheral blood analysis, 100–300 µL of blood was collected from the tail vein or from the heart following euthanasia and mixed with 10 µL of 500 mM EDTA to prevent clotting. Peripheral blood cell counts were determined using a Hemavet HV950 (Drew Scientific).

To analyze ubiquitylated proteins, 4 × 10^6^ cells were fixed with 500 µL of 1% paraformaldehyde (Thermo Fisher Scientific) in PBS for 15 min on ice after antibody staining for cell surface markers. Cells were resuspended in 200 µL of permeabilization buffer (1× PBS supplemented with 3% [v/v] fetal bovine serum [Sigma], 0.1% [m/v] saponin [Sigma]) and incubated for 5 min at room temperature. Next, cells were incubated with antiubiquitylated protein antibody (clone FK2; Millipore) at 1:5000 for 30 min at room temperature. This was followed by incubation with antimouse Alexa fluor 488 (Thermo Fisher Scientific) at 1:5000 for 30 min at room temperature. Cells were washed twice with permeabilization buffer and then resuspended in permeabilization buffer with 4 µg/mL DAPI. Samples were analyzed on a FACS Canto RUO flow cytometer.

### Colony formation assays

Single HSCs were sorted and seeded per well (96 wells) in Methocult GM M3434 (Stem Cell Technologies) supplemented with 10 ng/mL recombinant thrombopoietin (Peprotech) and 1× penicillin/streptomycin (Thermo Fisher Scientific). Colonies were assessed following 10–14 d of culture at 37°C. To measure colony cellularity, up to eight granulocyte macrophage (CFU-GM) and granulocyte, erythroid, macrophage, and megakaryocyte (CFU-GEMM) colonies were selected, and the average number of cells per colony was reported.

### Bone marrow reconstitution assays

Recipient mice (CD45.1/CD45.2 or CD45.1) were irradiated using an XRAD 320 X-ray irradiator (Precision X-Ray, Inc.) with two doses of 540 rad at least 3 h apart. Unfractionated bone marrow cells (5 × 10^5^) from donor (CD45.2) and competitor (CD45.1 or CD45.1/CD45.2) mice were mixed and injected intravenously through the tail vein. For secondary transplantation, 1 × 10^6^ unfractionated bone marrow cells from a single primary recipient at 16 wk following transplantation were injected intravenously into two to three lethally irradiated recipient mice. Every 4 wk until 16 wk after transplantation, 50–100 µL of blood was collected from the tail vein and mixed with 200 µL of 10 mM EDTA in PBS to prevent clotting. Cells were subjected to ammonium-chloride potassium chloride red blood cell lysis. Cells were stained with antibodies against CD45.1, CD45.2, Mac-1, Gr1, B220, and CD3 for donor cell engraftment analysis in the myeloid, B, and T cell lineages and detected using flow cytometry.

### Measuring the rate of protein synthesis

O-propargyl-puromycin (OP-Puro; 50 mg/kg body mass at pH 6.4–6.8 in PBS; Thermo Fisher Scientific) was injected intraperitoneally, and mice were euthanized for analysis 1 h later. Bone marrow was harvested, and 4 million cells were stained with antibodies against HSC surface markers as described above. After washing, cells were fixed in 0.5 mL of 1% paraformaldehyde (Thermo Fisher Scientific) in PBS for 15 min on ice. Cells were washed in 1× PBS and permeabilized in 200 µL of permeabilization buffer (1× PBS supplemented with 3% fetal bovine serum [Sigma], 0.1% saponin [Sigma]) for 5 min at room temperature. The azide-alkyne cycloaddition was performed using the Click-it cell reaction buffer kit (Life Technologies) and azide conjugated to Alexa fluor 555 (Thermo Fisher Scientific) at 5 µM final concentration. After 30 min, cells were washed twice in permeabilization buffer, resuspended in PBS with DAPI (4 µg/mL final concentration), and analyzed by flow cytometry.

### Protein extraction and Western blot

Twenty-thousand LSK cells were double-sorted into trichloroacetic acid (TCA; Sigma-Aldrich), and the TCA concentration was adjusted to 10%. Samples were incubated on ice and centrifuged at 16,000*g* for 15 min. The precipitates were washed twice with cold acetone and dried. Samples were solubilized in 9 M urea, 2% Triton X-100 (Fisher Scientific), and 1% DTT (Sigma Aldrich). NuPAGE LDS sample buffer (Life Technologies) was added and samples were heated for 10 min at 70°C. Proteins were separated on 4%–12% NuPAGE Bis-Tris polyacrylamide gels (Life Technologies) and transferred to a 0.2-µm PVDF membrane (Bio-Rad) by wet transfer and NuPAGE transfer buffer (Life Technologies). Antibodies were ARX (polyclonal; Abcam), Bmi1 (Cell Signaling Technology D42B3), Grp78/Bip (Cell Signaling Technology C50B12), phospho-eIF2α (Ser 51; polyclonal; Cell Signaling Technology), eIF2α (Cell Signaling Technology D7D3), Histone H3 (polyclonal; Abcam), K48-ub (polyclonal; Cell Signaling Technology), total ubiquitin (Cell Signaling Technology P4D1), p16^Ink4a^ (polyclonal; Santa Cruz Biotechnology), p19^Arf^ (polyclonal; Abcam), α-tubulin (Sigma DM1A), HRP-linked antirabbit IgG antibody (polyclonal; Cell Signaling Technology), and HRP-linked antimouse IgG antibody (polyclonal; Cell Signaling Technology). Signals were detected using SuperSignal West Pico or West Femto chemiluminescence kits (Thermo Fisher Scientific). In some cases, the SuperSignal Western blot enhancer (Thermo Fisher Scientific) was used. Blots were stripped with 0.2 M NaOH and blocked with 3% BSA/TBST before reprobing.

### RNA extraction

Cells (2000–5000) were sorted directly into Trizol LS (Thermo Fisher Scientific). Total RNA was extracted and reverse-transcribed using iScript reverse transcription supermix (Bio-Rad). Real-time PCR was performed using the iTaq Universal SYBR Green supermix (Bio-Rad) using a CFX384 real-time PCR machine (Bio-Rad). Transcript levels were normalized to Actin (*Actb*) using the Δ*Ct* method. The primers used for qPCR analysis are listed in Supplemental Table S3.

### Pyronin Y staining

Cells were collected via two methods that yielded similar results. In the first method, 20,000 LSK cells were sorted into tubes containing 200,000 B220^+^ sorted spleen cells. In the second method, following cKit enrichment and staining, cells were counted, and 250,000 cells were used for Pyronin Y staining. In both cases, cells were resuspended in HBSS staining media with 50 µg/mL verapamil (Sigma) and 30 µg/mL Hoescht (Thermo Fisher Scientific) and incubated for 45 min. Pyronin Y (Sigma) was added directly to a concentration of 1 µg/mL and cells were incubated for 15 min at 37°C. Cells were washed twice and resuspended in HBSS staining media with verapamil and immediately analyzed on a FACSAria II cytometer.

### Protein aggregation

Following HSC antibody staining, 4 × 10^6^ cells were fixed with 100 µL of BD Cytofix/Cytoperm buffer (BD Biosciences) for 15 min on ice. Cells were washed with 1× BD perm/wash buffer (BD Biosciences). Cells were then permeabilized with 100 µL of BD Cytoperm cell permeabilization plus buffer (BD Biosciences) for 5 min at room temperature and washed. Cells were then resuspended in 1× perm/wash buffer containing Proteostat dye (1:10,000; Enzo). Cells were incubated for 30 min at room temperature, washed, and resuspended in staining media containing 4 µg/mL DAPI followed by analysis on a FACSAria II cytometer.

### 5-bromo-2′-deoxyuridine Incorporation

Mice were injected intraperitoneally with 0.1 mg/g body mass 5-bromo-2′-deoxyuridine (BrdU) dissolved in PBS and then placed on drinking water containing 1 mg/mL BrdU for 72 h. Bone marrow from tibias, femurs, pelvis, and spine were harvested, and c-kit^+^ cells were enriched as described above. LSK stem and progenitor cells were sorted and mixed with 2 × 10^6^ carrier bone marrow cells from a mouse not treated with BrdU. Cells were fixed and stained for BrdU incorporation using the BD APC BrdU flow kit (BD Biosciences) following the manufacturer's instructions. For 10-d long-term BrdU incorporation experiments, mice were injected with 0.1 mg/g body mass BrdU and placed on drinking water containing 1 mg/mL BrdU for 10 d, with the water made fresh and replaced every 2 d. BrdU incorporation was analyzed using a FACSAria II cytometer.

### Single-cell RNA sequencing

Five-thousand double-sorted HSCs were collected per mouse, and HSCs from three mice were pooled to make one sample per genotype. Sorted cells were immediately processed for library preparation using the 10X Genomics Chromium Next GEM single-cell 3′ reagent kit v3.1 following the manufacturer's instructions. Libraries were quantified using the double-stranded DNA high-sensitivity assay kit (Thermo Fisher Scientific) on a Qubit fluorometer using high-sensitivity D1000 screen tapes and reagents (Agilent Technologies) with an Agilent 2200 TapeStation. Indexed libraries were sequenced on an Illumina NextSeq 500 platform using paired-end Illumina sequencing. For details related to the analysis of these data, see the Supplemental Material.

### Bulk RNA sequencing

Three-thousand HSCs per mouse (three control and three mutant) per time point were double-sorted into individual tubes containing 300 µL of RLT buffer (Qiagen RNAeasy micro kit), and RNA was purified according to the manufacturer's instructions. RNA integrity and concentration were assessed using a Pico Bioanalyzer. cDNA libraries were generated using the SMARTer stranded total RNA-seq sit v2-Pico input mammalian (Clonetech). Libraries were quantified using the double-stranded DNA high-sensitivity assay kit (Invitrogen) on a Qubit fluorometer and Agilent 2200 TapeStation. Indexed libraries were sequenced on an Illumina NextSeq 500 platform. For details related to the analysis of these data, see the Supplemental Material.

### Statistical methods

In each experiment, multiple mice were tested in multiple independent experiments performed on different days. Mice were allocated to experiments randomly and samples were processed in an arbitrary order. Sample sizes were not formally predetermined based on statistical power calculations but on our experience with the assays. The same samples were not repeatedly measured. Different replicates reflect samples obtained from different mice in most experiments. In competitive transplant assays, the same mice were repeatedly bled at different time points 4–16 wk after transplantation. Some of the data from 45- to 55-wk-old mice in Figure 1, B–D, were included in the data at the latest time point (>35 wk old) in Figure 1, E–I, because they were also from experiments in which we compared only *Bmi1*-deficient and control mice. For more details about the statistical tests that were performed, see the Supplemental Material.

### Accession numbers

Single-cell RNA sequencing data and bulk RNA sequencing data have been deposited in the NCBI Sequence Read Archive (BioProject IDs PRJNA834896 and PRJNA834838).

## Supplementary Material

Supplemental Material
