# Supplementary material for: Bmi1 suppresses protein synthesis and promotes proteostasis in hematopoietic stem cells
Source: Genes Dev. 2022 Aug 1;36(15-16):887–900. doi: 10.1101/gad.349917.122 (PMC9575696; doi:10.1101/gad.349917.122)
Supplement: Supplemental Material [file supp_36_15-16_887__DC1.html]

Bmi1 suppresses protein synthesis and promotes proteostasis in hematopoietic stem cells — Bmi1 suppresses protein synthesis and promotes proteostasis in hematopoietic stem cells — Supplemental Material 

# Bmi1 suppresses protein synthesis and promotes proteostasis in hematopoietic stem cells

## Supplemental Material

- Supplemental\_Data.pdf
