# Supplementary material for: Bmi1 suppresses protein synthesis and promotes proteostasis in hematopoietic stem cells
Source: Genes Dev. 2022 Aug 1;36(15-16):887–900. doi: 10.1101/gad.349917.122 (PMC9575696; doi:10.1101/gad.349917.122)
Supplement: Supplemental Material [file supp_gad.349917.122_Supplemental_Data.pdf]

Bmi1 suppresses protein synthesis and promotes proteostasis in hematopoietic stem cells

Rebecca J. Burgess[1], Zhiyu Zhao[1], Daisuke Nakada[2] and Sean J. Morrison[1,3,4*]

SUPPLEMENTAL MATERIAL

Supplemental Tables S1-S4

Supplemental Figures S1-S4

Supplemental Methods

Supplemental References

SUPPLEMENTAL TABLES

Supplemental Table S1. Cell populations analyzed by flow cytometry in this study

Cell Population	Abbreviation	Markers	Reference
Hematopoietic stem cells	HSC	$CD150^{+}CD48^{-}Lin^{-}Sca1^{+}c\text{-}kit^{+}$	(Kiel et al. 2005)
Multipotent progenitors	MPP	$CD150^{-}CD48^{-}Lin\text{-}Sca1^{+}c\text{-}kit^{+}$	(Kiel et al. 2005)
Hematopoietic stem and progenitor cells	LSK	$Lin\text{-}Sca1^{+}c\text{-}kit^{+}$	
Myeloid cells		Mac1 $(CD11b)^{+}Gr1^{+}$	
Erythroid cells		$Ter119^{+}$	
T cells		$CD3^{+}$	
proB		$B220^{+}IgM^{-}CD43^{+}CD24^{+}$	(Hardy et al. 1991)
preB		$B220^{+}IgM^{-}CD43^{-}$	(Hardy et al. 1991)
B220+IgM+		$B220^{+}IgM^{+}$	(Hardy et al. 1991)
B cells		$B220^{+}$	

Supplemental Table S2. **Bulk RNA seq analysis for control and *Mx1-Cre; Bmi1$^{fl/fl}$* HSCs, 12 weeks post pIpC treatment** (FDR<0.05, Fold Change>3)

Genes expressed at higher levels in *Bmi1* HSCs than control HSCs

Gene Name	Gene Description	Fold Change (mutant/control)	FDR
ARX	Aristaless related homeobox	34	0.019
CDKN2a	Cyclin-dependent kinase inhibitor	9.3	0.019
Tnni2	Troponin-1, skeletal, fast 2	7.0	0.019
Mcf2l	Mcf.2 transforming sequence-like	6.6	0.019
Pabpc1l	Poly(A) binding protein, cytoplasmic 1-like	4.1	0.019
Mgst1	Microsomal glutathione S-transferase	3.7	0.019

Genes expressed at lower levels in *Bmi1* HSCs than control HSCs

Gene Name	Gene Description	Fold Change (mutant/control)	FDR
Cldn5	Claudin 5	0.13	0.019
Mblac1	Metallo-beta-lactamase domain containing 1	0.16	0.019
Dera	Deoxyribose phosphate adolase	0.16	0.019
Tmcc1	Transmembrane and coiled coil domains 1	0.18	0.034
CD200	CD200 antigen	0.20	0.019
Itgb3	Integrin beta 3	0.20	0.019
Cdc42bpb	Cdc42 binding protein kinase beta	0.23	0.019
Slc5a2	Solute carrier family 5	0.24	0.019
Zfp119b	Zinc finger protein 119b	0.25	0.019
Egr1	Early growth response 1	0.28	0.019
Il22ra2	Interleukin 22 receptor, alpha 2	0.31	0.019
Etl4	Enhancer trap locus	0.32	0.034

Supplemental Table S3. Primers, sgRNAs, and donor oligos used in this study

Primer	Application	Sequences
Cre F	Genotyping	ATTGCTGTCACTTGGTCGTGGC
Cre R	Genotyping	GAAAATGCTTCTGTCCGTTTGC
Vav1-iCre F	Genotyping	AGATGCCAGGACATCAGGAACCTG
Vav1-iCre R	Genotyping	ATCAGCCACACCAGACACAGAGAT
Bmi1 floxed A	Genotyping	GCTAGCATTCCTGGTTTTGC
Bmi1 floxed B	Genotyping	GGCACAGTGATGAGGTGTTG
Bmi1 floxed C	Genotyping	CACGAGGTGCTTCTTTCCTC
$p16^{Ink4a}$ floxed A	Genotyping	GTTTTGGAGCAGCAGGGATT
$p16^{Ink4a}$ floxed B	Genotyping	CTATGTCAGATTTGGCTAGGGAGT
$p16^{Ink4a}$ floxed C	Genotyping	GTATGCTATACGAAGTTATTAGGTACTGC
$p19^{Arf}$ floxed A	Genotyping	TACTGCAGCCAGACCACTAGG
$p19^{Arf}$ floxed B	Genotyping	CGGAGATTGAGAAAGCGG
$p19^{Arf}$ floxed C	Genotyping	CGAGGAAATGTTGGGGCC
Cdkn2a A	Genotyping	GTGATCCCTCTACTTTTTCTTCTGACTT
Cdkn2a B	Genotyping	CGGAACGCAAATATCGCAC
Cdkn2a C	Genotyping	GAGACTAGTGAGACGTGCTACTTCCA
ARX floxed A	Genotyping	TGTGTGTGTTTCCCACCCTA
ARX floxed B	Genotyping	TCAATCAGGAGAAGGCTGGT
Actb F	QPCR	GCTCTTTTCCAGCCTTCCTT
Actb R	QPCR	CTTCTGCATCCTGTCAGCAA
$p16^{Ink4a}$ F	QPCR	CGAACTCTTTCGGTCGTACCC
$p16^{Ink4a}$ R	QPCR	CGAATCTGCACCGTAGTTGAGC
$p19^{Arf}$ F	QPCR	TGGTCACTGTGAGGATTCAGC
$p19^{Arf}$ R	QPCR	GTTGCCCATCATCATCACCTGG
ARX F	QPCR	TTTTCTAGGAGCAGCGGTGT
ARX R	QPCR	GTGTGGGCTGTCTCAGGA
Grp78 F	QPCR	CAGATCTTCTCCACGGCT
Grp78 R	QPCR	TGTCACTCGGAGAATACCAT
ARX 5' sgRNA	sgRNA	CATGTACTTGGGGTATGGAC
ARX 3' sgRNA	sgRNA	ACGTAGTTCAGGCTCAAACG
ARX exon 3	Donor oligo	TAAGTTTGAAGACTTATGGACTCAAGAATTCATCCC<br>CCCCCCACATCGCCACATTATATGATATAATATAAT<br>ATATGATATTTTATATCTGTCACCGTGCTAGGTTTTT<br>TTTTTTTTCTGTTTTCAAACAGTAGGCATTCTAGAG<br>GCAAAGATCAGTCTTACCCATGTACTTGGGGTATG<br>GGATCCATAACTTCGTATAATGTATGCTATACGAAG<br>TTATGACTGGAAGAAGCTGTCATGAAGGGGAAAGT<br>GGCTGAGAGAGGGTTACTGACCTGCCTGGATGTTG<br>CTTTCTTATAGGGAGGAGCTGGCCATGAGGCTGGA<br>CCTGACAGAGGCCCGAGTCCAGGTGAGCTCCAAG<br>AAGGGAAAAAGGAGGGCTAGGGTGGGTGAAGTGG<br>TGGTGGAGGGCCGCCCTTCAAATGGAAAGAAGAAA<br>ATCAAAATCTGAAAAGTAGACCCAAGCACTCACACA<br>GGAAAAGATATTTCAAAAGCAAGCTGCAAACTAGG<br>AAATAGGAGGAGAAGATTTCTGGCTCTGGAAGCTT<br>TAACGTAGTTCAGGCTCAAGGATCCATAACTTCGTA<br>TAATGTATGCTATACGAAGTTATACGGGGCTTTTCT

		AGTCCCTCAGAGTGCAACTCAGCCACCCCTGCCCC AACCTGGTACCCAAGGCCCCTATAGAAAACAATCC ACCAGCCTTCTCCTGATTGAAAT

Supplemental Table S4. Flow cytometry antibodies used in this study

Antibody	Clone
CD2	RM2-5
CD3	17A2
CD5	53-7.3
CD8a	53-6.7
CD45R (B220)	RA3-6B2
Gr1	RB6-8C5
Ter119	TER-119
c-kit	2B8
Sca1	D7; E13-161.7
CD150	TC15-12F12.2
CD48	HM48-1
IgM	II/41
CD43	R2/60
CD24	RRID
Mac-1 (Cd11b)	M1/70
CD45.1	A20
CD45.2	104

SUPPLEMENTAL FIGURES

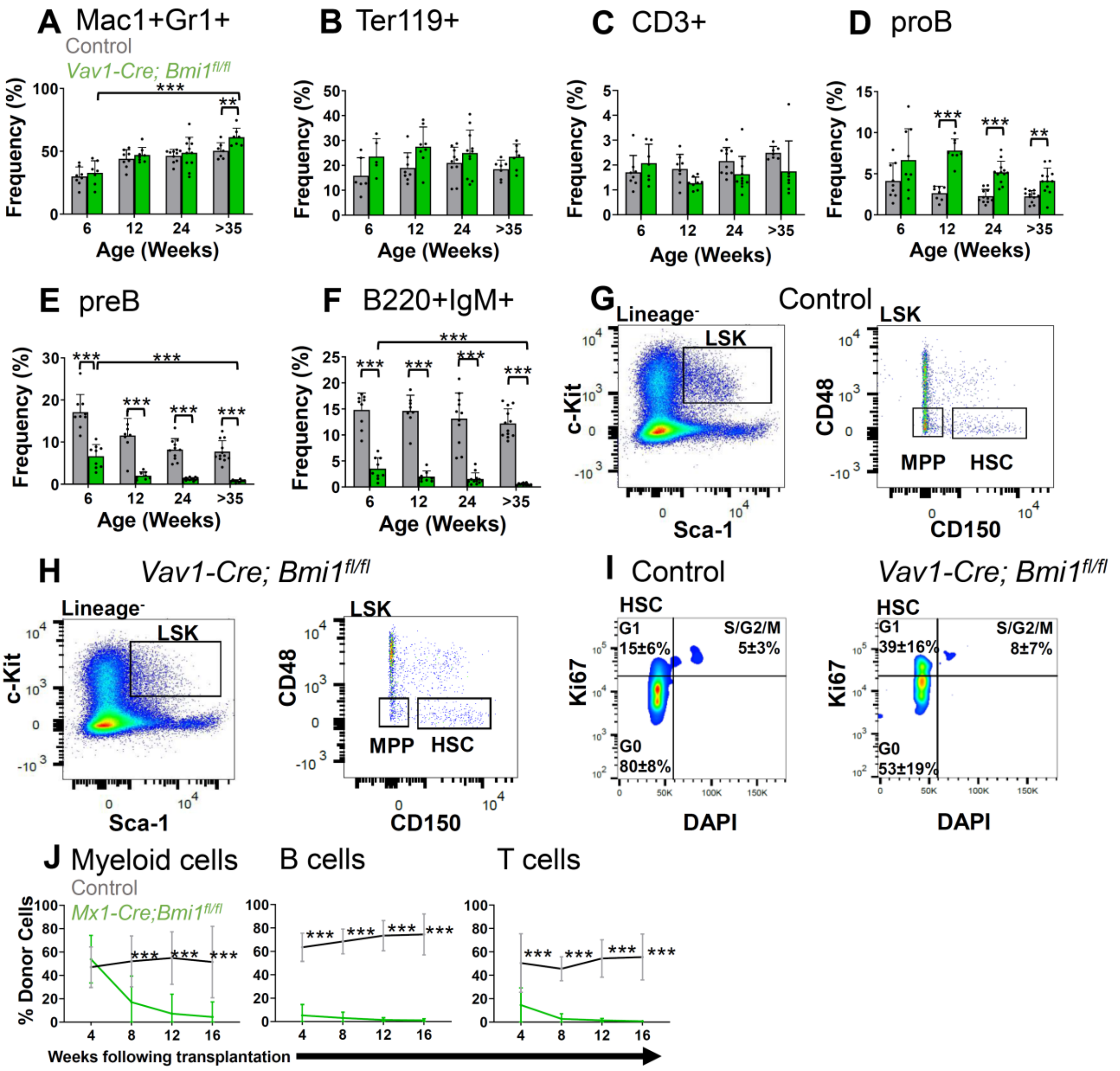

Supplemental Figure S1

Supplemental Figure S1: The effect of *Bmi1* deficiency on bone marrow hematopoiesis. (A-F) The frequencies of Mac-1$^+$Gr-1$^+$ myeloid cells (A), Ter119$^+$ erythroid cells (B), CD3$^+$ T cells (C), proB cells (D), preB cells (E), and B220$^+$IgM$^+$ B cells (F) in the bone marrow of *Vav1-Cre; Bmi1$^{fl/fl}$* and littermate control mice at 6-8 weeks, 12-16 weeks, 24-28 weeks, and >35 weeks of age (n= 5-

12 mice per genotype per time point from 6-11 independent experiments per time point). (G,H) Representative flow cytometry gates to identify HSCs and MPPs in control (G) and *Vav1-Cre; Bmi1*$^{fl/fl}$ (H) mice at 24 weeks of age. (I) Representative flow cytometric analyses of Ki67 and DAPI staining in HSCs from *Vav1-Cre; Bmi1*$^{fl/fl}$ and control mice at 35 weeks of age. (J) Donor Mac-1^{+}Gr-1^{+} myeloid cells, B220^{+} B cells and CD3^{+} T cells in the blood of mice competitively transplanted with *Mx1-Cre; Bmi1*$^{fl/fl}$ or control bone marrow cells (n=3 donors per genotype transplanted into a total of 12 or 14 recipient mice in 3 independent experiments). All data represent mean ± standard deviation (*p<0.05; **p<0.01; ***p<0.001) and each dot reflects a different mouse. Among *Bmi1* deficient groups, we only showed the statistical significance of differences between 6 weeks and 35 weeks.

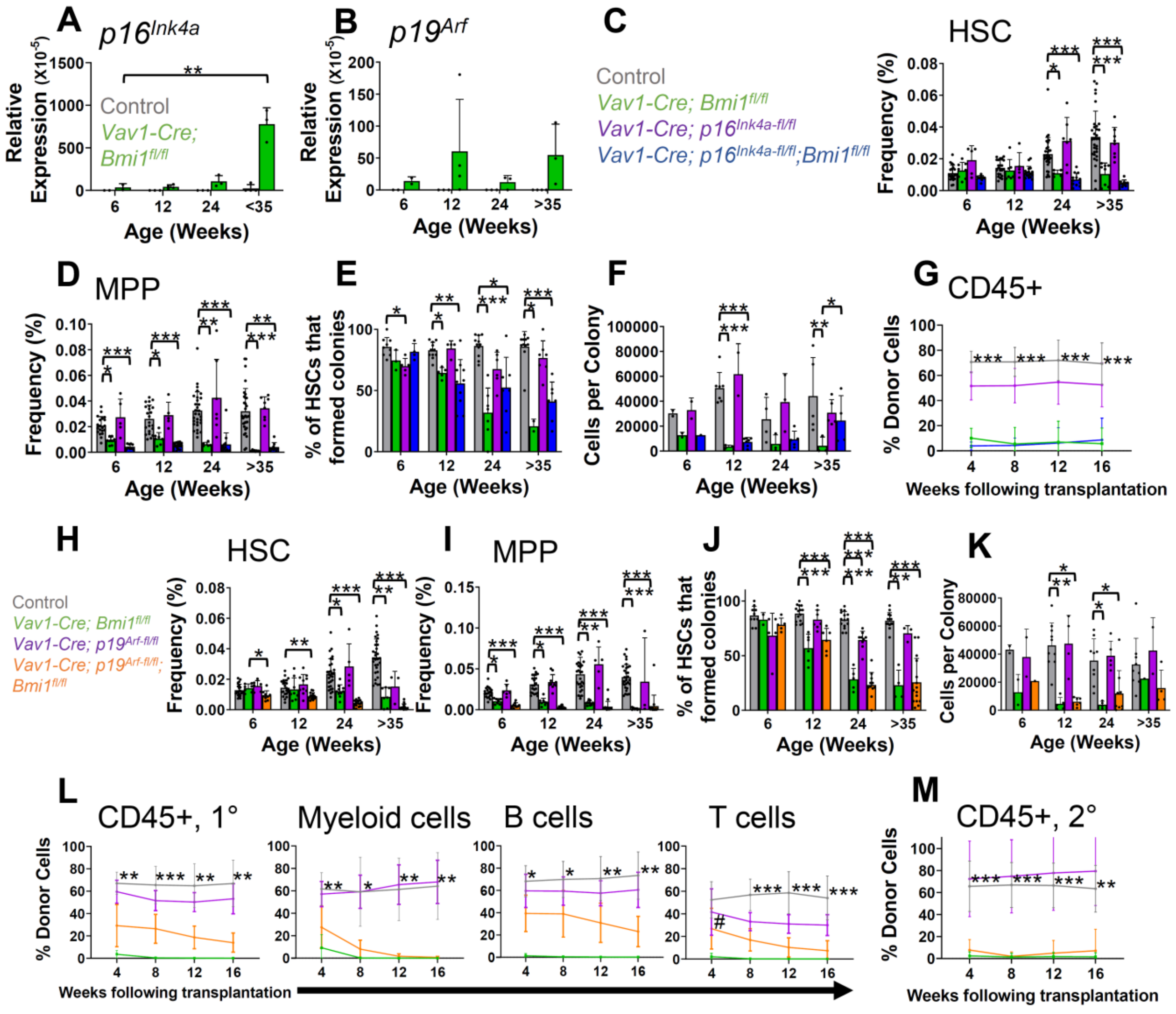

Supplemental Figure S2

Supplemental Figure S2: *p16^{ink4a}* deficiency or *p19^{Arf}* deficiency rescue the function of *Bmi1* deficient HSCs to a limited extent and do not rescue HSC depletion. (A-B) Quantitative RT-PCR analysis of *p16^{Ink4a}* (A) and *p19^{Arf}* (B) transcript levels in HSCs from *Vav1-Cre; Bmi1$^{fl/fl}$* and control mice (n=2-4 mice per genotype per time point in 2-3 independent experiments per time point). (C,D) Frequencies of HSCs (C) and MPPs (D) in the bone marrow of control, *Vav1-Cre; Bmi1$^{fl/fl}$*, *Vav1-Cre; p16$^{Ink4a-fl/fl}$* and *Vav1-Cre; p16$^{Ink4a-fl/fl}$; Bmi1$^{fl/fl}$* mice at 6-8 weeks, 12-16 weeks,

24-28 weeks and >35 weeks of age (n=5-32 mice per genotype per time point from 16-26 independent experiments per timepoint). (E,F) Percentage of HSCs that formed colonies in culture (E) and number of cells per colony (F) (n=1-11 mice per genotype per time point from 4-14 independent experiments per time point). (G) Donor CD45+ cells in the blood of mice competitively transplanted with bone marrow from control, *Vav1-Cre; Bmi1$^{fl/fl}$*, *Vav1-Cre; p16$^{Ink4a-fl/fl}$* or *Vav1-Cre; p16$^{Ink4a-fl/fl}$; Bmi1$^{fl/fl}$* mice (n=3 donor mice per genotype transplanted into a total of 10-14 recipients per genotype in 3 independent experiments). (H,I) Frequencies of HSCs (H) and MPPs (I) in bone marrow from control, *Vav1-Cre; Bmi1$^{fl/fl}$*, *Vav1-Cre; p19$^{Arf-fl/fl}$*, or *Vav1-Cre; p19$^{Arf-fl/fl}$; Bmi1$^{fl/fl}$* mice (n=3-31 mice per genotype per time point from 19-24 independent experiments per time point). (J, K) Percentage of HSCs that formed colonies (J) and number of cells per colony (K) (n=1-15 mice per genotype per time point from 4-17 independent experiments per time point). (L) Donor cell contribution to CD45$^+$ cells, Mac-1$^+$Gr-1$^+$ myeloid cells, B220$^+$ B cells, and CD3$^+$ T cells in the blood of mice competitively transplanted with donor bone marrow cells from control, *Vav1-Cre; Bmi1$^{fl/fl}$*, *Vav1-Cre; p19$^{Arf-fl/fl}$*, or *Vav1-Cre; p19$^{Arf-fl/fl}$; Bmi1$^{fl/fl}$* mice (n=4 donors per genotype transplanted into a total of 10-14 recipients per donor in 4 independent experiments). (M) Donor contribution to CD45$^+$ cells in the blood of secondary recipients transplanted with bone marrow cells from the primary recipients in (J) (n=3-4 donors per genotype transplanted into a total of 9-14 recipients in 3 independent experiments). All data represent mean ± standard deviation (*p<0.05; **p<0.01; ***p<0.001) and each dot reflects a different mouse. In the figure panels that show reconstitution assays, we only show the statistical significance of differences between mice reconstituted by control (*) or *Bmi1* deficient (#) cells versus double mutant cells (G, L, M).

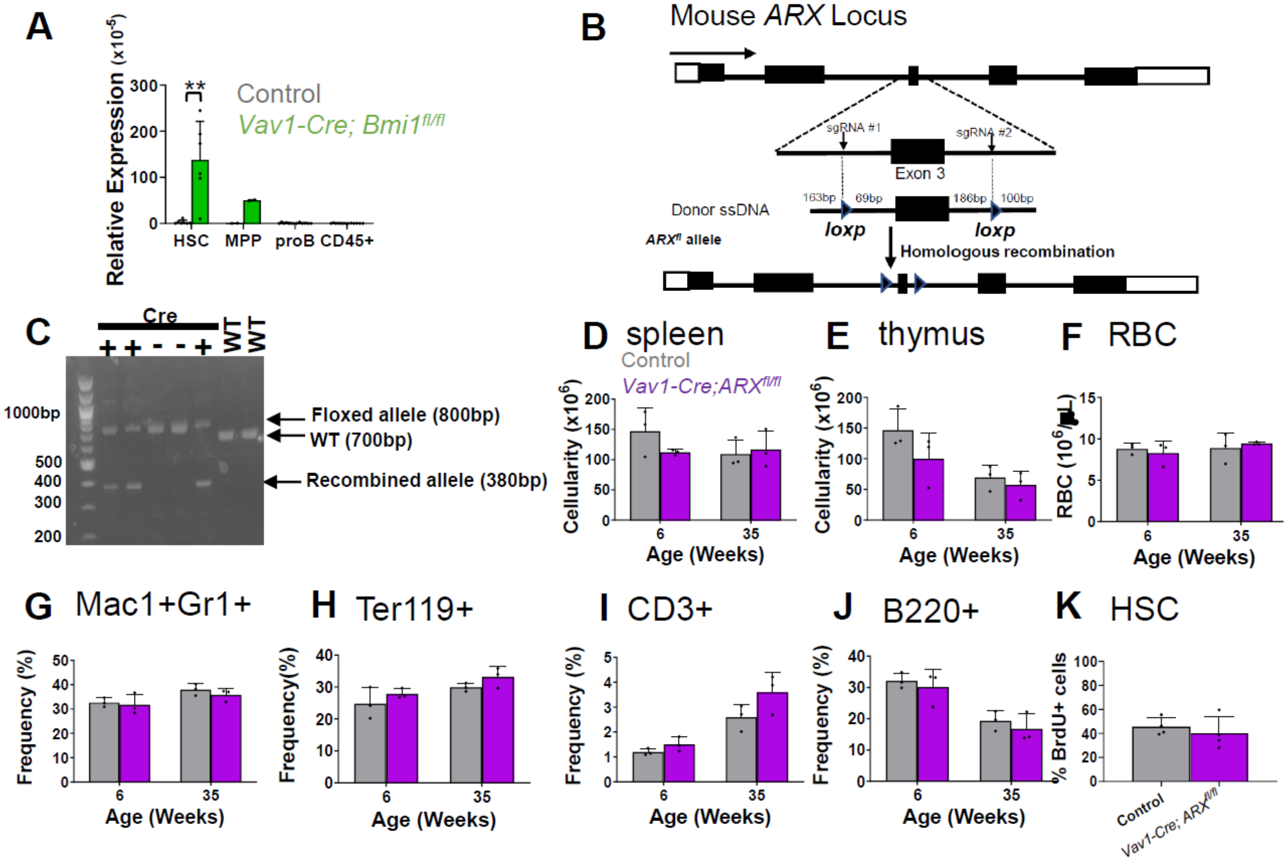

Supplemental Figure S3

Supplemental Figure S3: ARX is dispensable for normal hematopoiesis. (A) *ARX* expression in hematopoietic cells from *Vav1-Cre; Bmi1*$^{fl/fl}$ and control mice at 24-35 weeks of age (n=2-7 mice per genotype at 24-51 weeks of age from 4 experiments). (B) CRISPR gene-targeting strategy used to create the *ARX*^{fl} allele. The donor ssDNA contained loxp sequences flanking exon 3. The Cre-mediated deletion of exon 3 caused a frame-shift that eliminated the nuclear localization sequence and the β-catenin binding site of ARX. CRISPR/Cas9 mediated recombination was performed by injecting the donor ssDNA and 2 sgRNAs against endogenous sequences where the loxp sites were inserted. Correctly targeted founder mice were identified by long-range PCR with primers flanking the entire region targeted by the ssDNA followed by Sanger sequencing. The sequence of the donor ssDNA, sgRNAs, and primers used for genotyping are shown in

Supplemental Table S3. (C) PCR genotyping of the *ARX^{fl}* allele verifying recombination with *Vav1-Cre*. (D-F) spleen cellularity (D), thymus cellularity (E) and peripheral blood RBC cell counts (F) in *Vav1-Cre; $ARX^{fl/fl}$* and littermate control mice at 6-8 weeks and >35 weeks of age (n=3 mice per genotype per time point from 3 experiments per time point) (G-J) Frequencies of Mac-1$^+$Gr-1$^+$ myeloid cells (G), Ter119$^+$ erythroid cells (H), CD3$^+$ T cells (I), and B220$^+$ B cells (J) in the bone marrow of *Vav1-Cre; $ARX^{fl/fl}$* and control mice. (K) Incorporation of a 72-hour pulse of BrdU into HSCs from *Vav1-Cre; $ARX^{fl/fl}$* and control mice at 24-28 weeks of age (n=4 mice per genotype from 3 experiments). All data represent mean ± standard deviation (*p<0.05; **p<0.01; ***p<0.001) and each dot represents a different mouse.

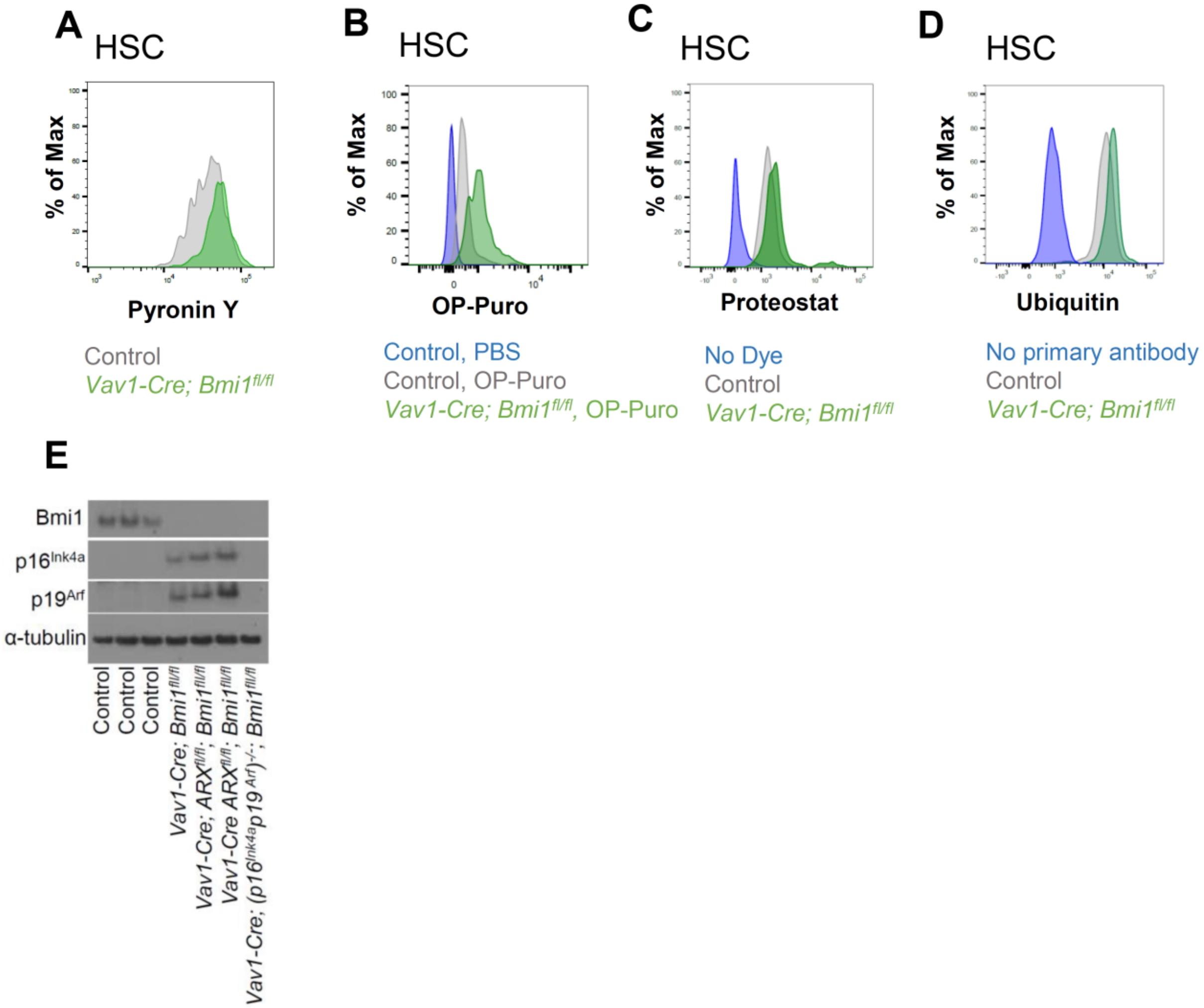

Supplemental Figure S4

Supplemental Figure S4: Bmi1 suppresses protein synthesis and promotes proteostasis in HSCs. (A-D) Histograms of flow cytometric analyses of Pyronin Y staining (A), OP-Puro incorporation (B), Proteostat dye staining (C), and poly and mono-ubiquitylated protein levels (D) in HSCs from 35 week old *Vav1-Cre; Bmi1$^{fl/fl}$* and littermate control mice. Each histogram is from a single experiment but the results are representative of 2 to 6 independent experiments per panel. (E) Western blot analysis of the levels of Bmi1, p16^{Ink4a}, p19^{Arf} and α-tubulin in the bone marrow of control, *Vav1-Cre; Bmi1$^{fl/fl}$*, *Vav1-Cre; ARX$^{fl/fl}$; Bmi1$^{fl/fl}$* and *Vav1-Cre; (p16^{Ink4a}p19^{Arf})$^{-/-}$; Bmi1$^{fl/fl}$* mice at 24-35 weeks of age.

SUPPLEMENTAL METHODS

Analysis of single cell RNA sequencing data

Raw sequencing reads from individual samples were demultiplexed, counted, and aggregated using 10x Genomics Cell Ranger 4.0.0 with the mouse mm10 reference genome. Stressed or dying cells with >5% of their transcripts from mitochondrial genes were excluded from analysis using R 4.0.2 with Seurat 3.2 on Cell Ranger's filtered feature barcode matrix of Unique Molecular Identifier (UMI) counts. Filtered samples were then normalized and integrated using Seurat's variance stabilizing transformations for single cell UMI data (SCTransform). HSC clustering was performed using Seurat 3.2 (Stuart et al. 2019) with and without cell cycle scoring and regression (Tirosh et al. 2016). LT-HSC and ST-HSC signature scores were calculated using published gene lists (Pei et al. 2020) and scoring differences between cells were tested by a linear mixed-effects model followed by the False Discovery Rate method for multiple comparisons adjustment using R with the Ime4 package. Differential gene expression analysis was performed by Wilcoxon rank sum tests using Seurat, and gene set enrichment analyses were performed using GSEA 4.1.0 (Mootha et al. 2003; Subramanian et al. 2005) on the pre-ranked, log-transformed gene fold changes. GO Term enrichment of cell marker genes was performed using clusterProfiler 3.18.1(Yu et al. 2012).

Analysis of bulk RNA sequencing data

RNAseq data were analyzed based on the Tuxedo protocol (Trapnell et al. 2012; Pertea et al. 2016), and the quality of raw reads were assessed by FASTQC 0.11 (Andrews 2010). Raw reads were trimmed using TrimGalore 0.6 and mapped to the Ensembl GRCm38 mouse reference genome using TopHat2 (Kim et al. 2013) with Bowtie2. Mapped reads were quality-filtered using SAMtools 1.9 (Li et al. 2009) and quantified using Cufflinks 2 (Trapnell et al. 2010; Trapnell et al. 2012). Quantified mapped reads were normalized to fragments per 10000 exonic bases per million mapped reads (FPKMs) and gene expression levels measured using Cuffnorm. Differential gene

expression was assessed using Cuffdiff. Expressed ribosome genes were determined as protein coding genes with FPKM>1. The correlation among genes that were differentially expressed between control and *Bmi1* deficient HSCs in the single cell RNA sequencing and bulk RNA sequencing was tested using only genes with detectable expression in both datasets. The statistical significance of the correlation was assessed using a Pearson correlation test.

Supplemental statistical methods

To analyze the statistical significance of differences among groups, we first tested if data were normally distributed and if variance was similar among groups. To test for normality, we performed the D'Agostino Omnibus test when n≥20 or the Shapiro–Wilk test for smaller sample numbers. To test for significant differences in variability among groups, we performed an *F*-test (for experiments with two groups) or Levene's median test (more than two groups). When the data significantly deviated from normality or variability significantly differed among groups, we log2-transformed the data and tested again for normality and variability. If the transformed data did not significantly deviate from normality and equal variability, we performed parametric tests on the transformed data. If log2-transformation was not possible or the transformed data still significantly deviated from normality or equal variability, we performed non-parametric tests on the non-transformed data. The dagoTest and shapiroTest functions of the fBasics package were used to perform the normality tests, and the leveneTest function of the Companion to Applied Regression package was used to perform the Levene's median test for variance.

When data or log2-transformed data were normal and equally variable, statistical analyses were performed using Student's t-tests (when there were two groups), one-way ANOVAs (when there were more than two groups), or two-way ANOVAs (when there were two or more groups with multiple tissues or cell populations or time points). When the data or log2-transformed data were normal but unequally variable, statistical analyses were performed using Welch's t-tests (when there were two groups) or Welch's one-way ANOVAs (when there were more

than two groups). When the data and log2-transformed data were abnormal or unequally variable, statistical analysis was performed using Mann-Whitney tests (when there were two groups), Kruskal-Wallis tests (when there were more than two groups. P-values from multiple comparisons were adjusted using Tukey's test (when there were more than two groups and all the comparisons were of interest), Dunnett's method (when there were more than two groups and comparisons between the control and all other groups were of interest) or Sidak's method (when there were more than two groups and planned comparisons) after ANOVAs, Dunnett's T3 method after Welch's one-way ANOVAs, or Dunn's method after Kruskal-Wallis tests. Holm-Sidak's method was used to adjust comparisons involving multiple cell populations with two groups after multiple tissues, cell populations or time points with two groups after multiple Student's t-tests or Mann-Whitney tests. Survival data was analyzed using the log-rank (Mantel-Cox) test. All statistical tests were two-sided. All data represent mean ± standard deviation. Statistical tests were performed using GraphPad Prism V9.1.0 or R 4.0.2.

Supplemental References

Andrews S. 2010. A Quality Control Tool for HIgh Throughput Sequence Data. http://www.bioinformatics.babraham.ac.uk/projects/fastqc/.

Hardy RR, Carmack CE, Shinton SA, Kemp JD, Hayakawa K. 1991. Resolution and characterization of pro-B and pre-pro-B cell stages in normal mouse bone marrow. *J Exp Med* **173**: 1213-1225.

Kiel MJ, Yilmaz OH, Iwashita T, Yilmaz OH, Terhorst C, Morrison SJ. 2005. SLAM family receptors distinguish hematopoietic stem and progenitor cells and reveal endothelial niches for stem cells. *Cell* **121**: 1109-1121.

Kim D, Pertea G, Trapnell C, Pimentel H, Kelley R, Salzberg SL. 2013. TopHat2: accurate alignment of transcriptomes in the presence of insertions, deletions and gene fusions. *Genome Biol* **14**: R36.

Li H, Handsaker B, Wysoker A, Fennell T, Ruan J, Homer N, Marth G, Abecasis G, Durbin R, Genome Project Data Processing S. 2009. The Sequence Alignment/Map format and SAMtools. *Bioinformatics* **25**: 2078-2079.

Mootha VK, Lindgren CM, Eriksson KF, Subramanian A, Sihag S, Lehar J, Puigserver P, Carlsson E, Ridderstrale M, Laurila E et al. 2003. PGC-1alpha-responsive genes involved in oxidative phosphorylation are coordinately downregulated in human diabetes. *Nat Genet* **34**: 267-273.

Pei W, Shang F, Wang X, Fanti AK, Greco A, Busch K, Klapproth K, Zhang Q, Quedenau C, Sauer S et al. 2020. Resolving Fates and Single-Cell Transcriptomes of Hematopoietic Stem Cell Clones by PolyloxExpress Barcoding. *Cell Stem Cell* **27**: 383-395 e388.

Pertea M, Kim D, Pertea GM, Leek JT, Salzberg SL. 2016. Transcript-level expression analysis of RNA-seq experiments with HISAT, StringTie and Ballgown. *Nat Protoc* **11**: 1650-1667.

Stuart T, Butler A, Hoffman P, Hafemeister C, Papalexi E, Mauck WM, 3rd, Hao Y, Stoeckius M, Smibert P, Satija R. 2019. Comprehensive Integration of Single-Cell Data. *Cell* **177**: 1888-1902 e1821.

Subramanian A, Tamayo P, Mootha VK, Mukherjee S, Ebert BL, Gillette MA, Paulovich A, Pomeroy SL, Golub TR, Lander ES et al. 2005. Gene set enrichment analysis: a knowledge-based approach for interpreting genome-wide expression profiles. *Proc Natl Acad Sci U S A* **102**: 15545-15550.

Tirosh I, Izar B, Prakadan SM, Wadsworth MH, 2nd, Treacy D, Trombetta JJ, Rotem A, Rodman C, Lian C, Murphy G et al. 2016. Dissecting the multicellular ecosystem of metastatic melanoma by single-cell RNA-seq. *Science* **352**: 189-196.

Trapnell C, Roberts A, Goff L, Pertea G, Kim D, Kelley DR, Pimentel H, Salzberg SL, Rinn JL, Pachter L. 2012. Differential gene and transcript expression analysis of RNA-seq experiments with TopHat and Cufflinks. *Nat Protoc* **7**: 562-578.

Trapnell C, Williams BA, Pertea G, Mortazavi A, Kwan G, van Baren MJ, Salzberg SL, Wold BJ, Pachter L. 2010. Transcript assembly and quantification by RNA-Seq reveals unannotated transcripts and isoform switching during cell differentiation. *Nat Biotechnol* **28**: 511-515.

Yu G, Wang LG, Han Y, He QY. 2012. clusterProfiler: an R package for comparing biological themes among gene clusters. *OMICS* **16**: 284-287.
